# Trends and patterns of disparities in diabetes and chronic kidney disease mortality among US counties, 1980–2014

**DOI:** 10.1186/s12963-022-00285-4

**Published:** 2022-02-22

**Authors:** Ali H. Mokdad, Laura Dwyer-Lindgren, Amelia Bertozzi-Villa, Rebecca W. Stubbs, Chloe Morozoff, Shreya Shirude, Sam B. Finegold, Charlton Callender, Mohsen Naghavi, Christopher J. L. Murray

**Affiliations:** 1 Institute for Health Metrics and Evaluation, University of Washington, 3980 15th Ave NE, Seattle, WA 98195 USA; 2 Department of Health Metrics Sciences, University of Washington, Seattle, USA

**Keywords:** Diabetes, Chronic kidney disease, Disparities, Mortality

## Abstract

**Introduction:**

Diabetes and chronic kidney diseases are associated with a large health burden in the USA and globally.

**Objective:**

To estimate age-standardized mortality rates by county from diabetes mellitus and chronic kidney disease.

**Design and setting:**

Validated small area estimation models were applied to de-identified death records from the National Center for Health Statistics (NCHS) and population counts from the census bureau, NCHS, and the Human Mortality Database to estimate county-level mortality rates from 1980 to 2014 from diabetes mellitus and chronic kidney disease (CKD).

**Exposures:**

County of residence.

**Main outcomes and measures:**

Age-standardized mortality rates by county, year, sex, and cause.

**Results:**

Between 1980 and 2014, 2,067,805 deaths due to diabetes were recorded in the USA. The mortality rate due to diabetes increased by 33.6% (95% UI: 26.5%–41.3%) between 1980 and 2000 and then declined by 26.4% (95% UI: 22.8%–30.0%) between 2000 and 2014. Counties with very high mortality rates were found along the southern half of the Mississippi river and in parts of South and North Dakota, while very low rates were observed in central Colorado, and select counties in the Midwest, California, and southern Florida. A total of 1,659,045 deaths due to CKD were recorded between 1980 and 2014 (477,332 due to diabetes mellitus, 1,056,150 due to hypertension, 122,795 due to glomerulonephritis, and 2,768 due to other causes). CKD mortality varied among counties with very low mortality rates observed in central Colorado as well as some counties in southern Florida, California, and Great Plains states. High mortality rates from CKD were observed in counties throughout much of the Deep South, and a cluster of counties with particularly high rates was observed around the Mississippi river.

**Conclusions and relevance:**

This study found large inequalities in diabetes and CKD mortality among US counties. The findings provide insights into the root causes of this variation and call for improvements in risk factors, access to medical care, and quality of medical care.

**Supplementary Information:**

The online version contains supplementary material available at 10.1186/s12963-022-00285-4.

## Introduction

Diabetes mellitus accounted for 77.7 thousand deaths (2.6% of all deaths) and 4.46 million disability-adjusted life years (DALYs) in 2019 in the USA [1, 2]. Diabetes prevalence has increased rapidly in the USA in recent decades, reaching 11.8% in 2019 [2, 3]. Diabetes is associated with several diseases including chronic kidney disease (CKD). CKD was the 6th leading cause of death in 2019, accounting for 3.6% of all deaths [2]. In 1990, CKD, which is preventable by adequate medical care, was the 14th leading cause of death, accounting for 1.5% of all deaths [2].

The prevalence of diabetes has increased despite previous calls for action**.** Recent data from the Behavioral Risk Factor Surveillance System (BRFSS), a large state-based surveillance system, show that the self-reported prevalence of diagnosed diabetes in 2020 was 10.6% among adults aged 18 or older. West Virginia had the highest prevalence (15.7%) and Alabama 14.8%, while District of Columbia (7.5%) and Colorado (7.6%) had the lowest rates [4].

Obesity increased in all states from 1990 to 2020 [4]. Obesity is a major risk factor for type 2 diabetes, and there is a significant association between weight gain and diabetes incidence [5–7]. The prevalence of obesity it is likely to continue to rise in the years ahead unless effective interventions are implemented. Furthermore, diabetes is associated with a high medical cost [8]. Behavioral and metabolic risk factors such as poor diet and lack of physical activity are also risk factors for type 2 diabetes [9, 10]. Therefore, diabetes is expected to increase rapidly in the coming decades due to aging and growth of the US population, poor diet, obesity, and low physical activity [11, 12].

Diabetes mortality rates vary by states and counties in the USA, masking disparities [13, 14]. Knowing where the “hotspots” are will guide health professionals to target high-risk communities using limited resources. This study presents trends in mortality from diabetes and chronic kidney disease for US counties from 1980 to 2014.

## Methods

The methods used for this analysis were previously reported in detail elsewhere and are described briefly here [15]. This research received institutional review board approval from the University of Washington. Informed consent was not required because the study used de-identified data and was retrospective.

### Data

This analysis used de-identified death records from the National Center for Health Statistics (NCHS) [16] and population counts from the census bureau [17], NCHS [18–20], and the Human Mortality Database [21]. Deaths and population were tabulated by county, age group (0, 1–4, 5–9, …, 75–79, and ≥ 80), sex, year, and cause. County-level information on levels of education, income, race/ethnicity, Native American reservations, and population density derived from data provided by the census bureau and NCHS was utilized as covariates (more detail on these data sources is available in Additional file 1: eTable S1 in the supplement). These variables were selected based on data availability because we expect that these variables are likely to be predictive of mortality across a range of causes. In a small number of cases, counties were combined to ensure historically stable units of analysis (Additional file 1: eTable S2).

### Cause list and garbage redistribution

The study used the cause list developed for the Global Burden of Diseases, Injuries, and Risk Factors Study (GBD) [22]. This cause list is arranged hierarchically in four levels, and within each level the list is exhaustive and mutually exclusive. Additional file 1: eTable S3 in the supplement lists all causes in the GBD cause list and the ICD9 and ICD10 codes that correspond to each cause. The focus of this study was on diabetes mellitus and chronic kidney disease. Chronic kidney disease was also sub-divided into chronic kidney disease due to diabetes mellitus, chronic kidney disease due to hypertension, chronic kidney disease due to glomerulonephritis, and chronic kidney disease due to other causes. Although the focus of this study was diabetes mellitus and chronic kidney disease, all causes of death in the GBD cause list were analyzed concurrently.

Previous studies have documented the existence of insufficiently specific or implausible causes of death used in death registration data that may lead to misleading geographic and temporal patterns [23]. Algorithms developed for the GBD were used to reallocate deaths assigned one of these “garbage codes” to plausible alternatives [22]. First, plausible target causes were assigned to each garbage code or group of garbage codes. Second, deaths were reassigned to specified target codes according to proportions derived in one of four ways: (1) published literature or expert opinion; (2) regression models; (3) according to the proportions initially observed among targets; and (4) for HIV/AIDS specifically, by comparison with years before HIV/AIDS became widespread.

### Small area models

The number of deaths observed in a given county, year, age, and sex is typically small, and the directly observed mortality rates at this level are often highly unstable. We use a small area estimation model to stabilize these estimates by “borrowing strength” across counties, time periods, age groups, and from external information (covariates). This model has been previously validated and shown to perform well even for counties with relatively small populations. [15]

Bayesian, spatially explicit mixed effects regression models were estimated for each cause in the GBD hierarchy, separately for males and females. The model for each cause was specified as:

$$\begin{aligned} D_{j,t,a} &\sim {\text{Poisson}}\left( {m_{j,t,a} \cdot P_{j,t,a} } \right) \\ \log \left( {m_{j,t,a} } \right) &= \beta_{0} + {\varvec{\beta}}_{1} \cdot {\varvec{X}}_{j,t} + \gamma_{1,a,t} + \gamma_{2,j} \\ &\quad+ (\gamma_{3,j} \cdot t + \gamma_{4,j,t} ) + \left( {\gamma_{5,j} \cdot a + \gamma_{6,j,a} } \right) \\ \end{aligned}$$

where $D_{j,t,a}$, $P_{j,t,a}$ and $m_{j,t,a}$ are the number of deaths, the population, and the underlying mortality rate, respectively, for county *j*, year *t*, and age group *a.* The model for $m_{j,t,a}$ contained six components: an intercept ($\beta_{0}$), fixed covariate effects (${\varvec{\beta}}_{1}$), random age-time effects ($\gamma_{1,a,t}$), random spatial effects ($\gamma_{2,j}$), random space–time effects ($\gamma_{3,j}$ and $\gamma_{4,j,t}$), and random space-age effects ($\gamma_{5,j}$ and $\gamma_{6,j,a}$). The model incorporated seven covariates: the proportion of the adult population who graduated high school, the proportion of the population that is Hispanic, the proportion of the population that is Black, the proportion of the population that is a race other than Black or White, the proportion of a county that is contained within a state or federal Native American reservation, the median household income, and the population density. ${\varvec{\gamma}}_{1}$, ${\varvec{\gamma}}_{2}$, ${\varvec{\gamma}}_{3}$, and ${\varvec{\gamma}}_{5}$ were assumed to follow conditional autoregressive distributions, which allow for smoothing over adjacent age groups and years (${\varvec{\gamma}}_{1}$) or counties (${\varvec{\gamma}}_{2}$, ${\varvec{\gamma}}_{3}$, and ${\varvec{\gamma}}_{5}$). [24, 25]. ${\varvec{\gamma}}_{4}$ and ${\varvec{\gamma}}_{6}$ were assumed to follow independent mean-zero normal distributions.

Models were fit using the Template Model Builder Package [26] in the R version 3.2.4 statistical software (R Foundation for Statistical Computing). One thousand draws of m_*j,t,a*_ were taken from the approximated posterior distribution. These draws were raked [27] (i.e., scaled along multiple dimensions) to ensure consistency between levels of the cause hierarchy and to ensure consistency with national estimates from the GBD [22]. After raking, age-standardized mortality rates were calculated using the US 2010 census population as the standard, and years of life lost (YLLs) were calculated for each age group by multiplying the mortality rate by population by life expectancy at the average age at death in the reference life table used in the GBD [22] and then summing across all ages. Point estimates were calculated from the mean of all draws, and 95% uncertainty intervals were calculated from the 2.5th and 97.5th percentiles. Changes over time were considered statistically significant if the posterior probability of an increase (or decrease) was ≥ 95%. Code for fitting the small area models is available from the authors upon request.

## Results

Deaths, years of life lost, and age-standardized mortality rates at the national level, and the distribution of age-standardized mortality rates at the county level by cause in 2014 are presented in Table 1. Chronic kidney disease had the higher mortality rate in 2014 (22.4 [95% UI: 21.4–23.3] deaths per 100,000 population) followed by diabetes (19.7 [95% UI: 18.9–20.6] deaths per 100,000 population).

**Table 1:** National deaths, years of life lost (YLLs), and age-standardized mortality rate; and distribution of age-standardized mortality rates at the county level (2014)

Cause of death	National deaths, YLLs, and mortality rate			County-level mortality rates						
	Deaths, no. in thousands	YLLs, no. in Thousands	Mortality rate, no. of deaths/100,000 population	No. of deaths/100,000 population					90th*–*10th percentiles, no. of deaths/100,000 population^a^	Ratio of 90th–10th percentile, ratio^b^
				Minimum	10th percentile	Median	90th percentile	Maximum		
Diabetes mellitus	66.2 (63.6–69.1)	1182.5 (1133.3–1231.1)	19.7 (18.9–20.6)	2.4	13.8	21.3	33.8	118.7	20.0	2.4
Chronic kidney disease	75.0 (71.7–78.1)	1067.8 (1028.3–1104.6)	22.4 (21.4–23.3)	4.9	16.2	24.1	35.1	70.2	18.9	2.2
…Due to diabetes mellitus	29.6 (27.1–33.3)	496.1 (461.0–540.9)	8.8 (8.1–9.9)	2.1	6.5	9.8	14.2	29.4	7.7	2.2
…Due to hypertension	38.2 (34.9–41.0)	489.4 (449.8–525.0)	11.4 (10.4–12.3)	2.1	7.5	11.9	18.5	41.3	11.0	2.5
…Due to glomerulonephritis	6.6 (5.7–7.5)	75.6 (68.1–83.5)	2.0 (1.7–2.2)	0.6	1.5	2.0	2.7	6.2	1.2	1.8
…Due to other causes	0.6 (0.4–0.7)	6.7 (5.6–8.0)	0.2 (0.1–0.2)	0.1	0.1	0.2	0.4	2.2	0.2	2.7

YLLs, years of life lost

^a^Measure of absolute geographic inequality

^b^Measure of relative geographic inequality

### Diabetes

**Fig. 1:**
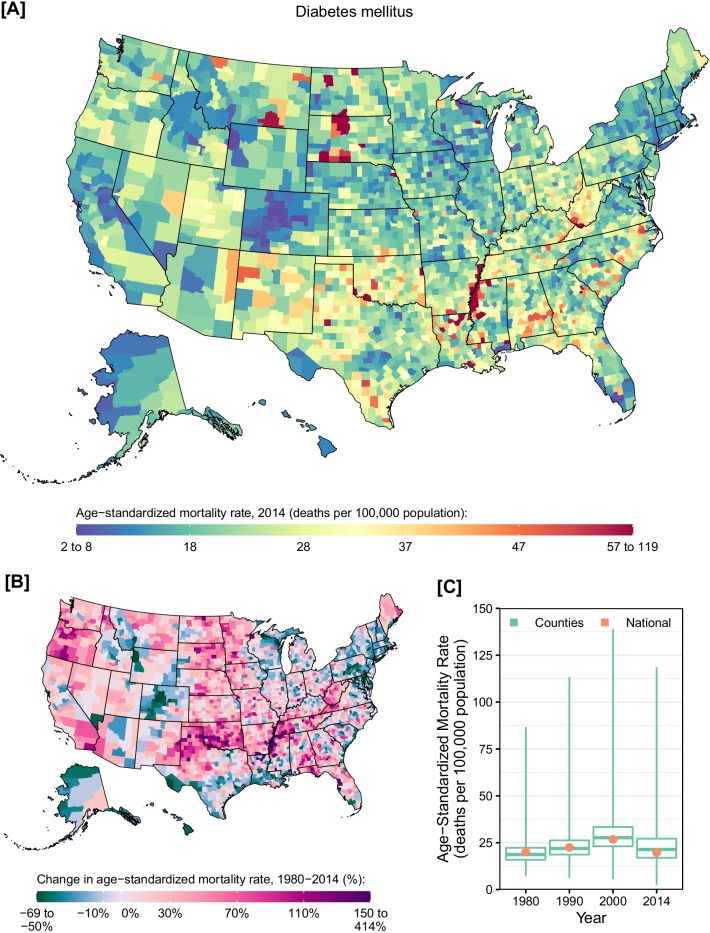
County-level mortality from diabetes mellitus. **A** Age-standardized mortality rate in 2014; **B** Relative change in the age-standardized mortality rate between 1980 and 2014; **C** Age-standardized mortality rate in 1980, 1990, 2000, and 2014. In panels **A** and **B**, the color scale is truncated at approximately the 1st and 99th percentiles as indicated by the range given in the color scale. In panel **C**, the boxes indicate the 25th, 50th, and 75th percentiles across all counties, while the lines indicate the full range across counties and the dots indicate the national-level rate

Between 1980 and 2014, 2,067,805 deaths due to diabetes were recorded in the USA. The mortality rate due to diabetes increased by 33.6% (95% UI: 26.5–41.3%) between 1980 and 2000 and then declined by 26.4% (95% UI: 22.8–30.0%) between 2000 and 2014. The age-standardized mortality rate from diabetes was 20.1 (95% UI: 19.2–21.0), 22.4 (95% UI: 21.8–23.1), 26.8 (95% UI: 26.1–27.6), and 19.7 (95% UI: 18.9–20.6) deaths per 100,000 population in 1980, 1990, 2000, and 2014, respectively. Counties with very high mortality rates were found along the southern half of the Mississippi river and in parts of South and North Dakota (Fig. 1). On the other hand, counties with very low rates were observed in central Colorado, and select counties in the Midwest, California, and southern Florida. Among counties, the lowest estimated mortality rate in 2014 was observed in Summit County, Colorado (2.4 [95% UI: 1.9–2.8] deaths per 100,000 population), while the highest was observed in Oglala Lakota County, South Dakota (118.7 [95% UI: 106.2–132.1] deaths per 100,000 population). Most counties experienced an increase in the mortality rate due to diabetes between 1980 and 2014 (65.9%; statistically significant in 51.6%), but counties where the mortality rate declined were found in most states. Similar geographic patterns were observed for males and females in 2014 (Additional file 1: eFigs. S1 and S2); however, the mortality rate from diabetes increased by 12.5% (95% UI: 5.4–20.2%) among males and decreased by 14.5% (95% UI: 7.0–22.7%) among females between 1980 and 2014.

### Chronic kidney disease

**Fig. 2:**
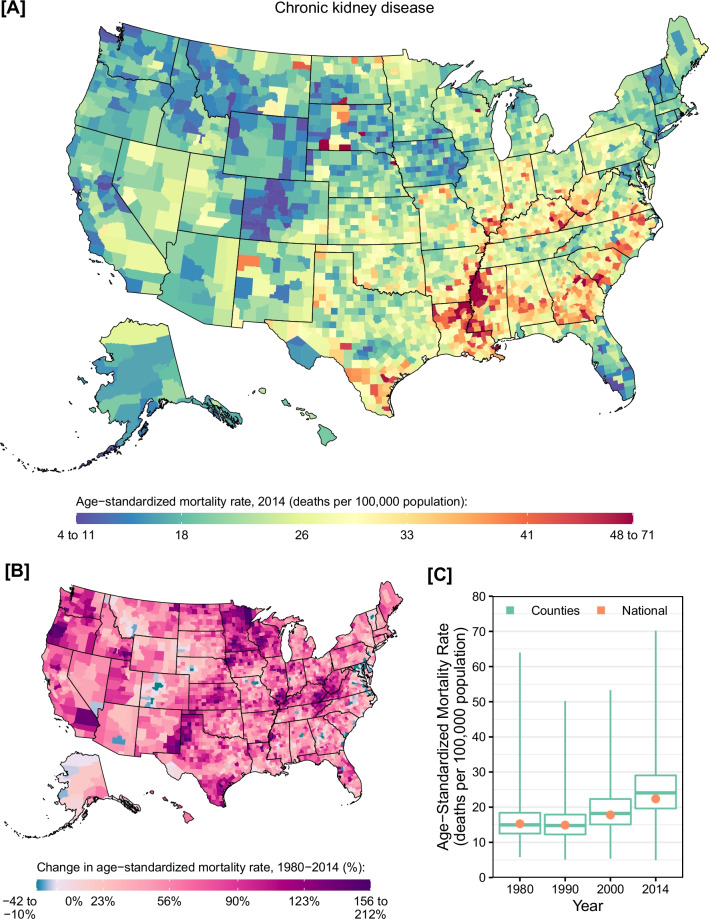
County-level mortality from chronic kidney disease. **A** Age-standardized mortality rate in 2014; **B** relative change in the age-standardized mortality rate between 1980 and 2014; **C** age-standardized mortality rate in 1980, 1990, 2000, and 2014. In panels **A** and **B**, the color scale is truncated at approximately the 1st and 99th percentiles as indicated by the range given in the color scale. In panel **C**, the boxes indicate the 25th, 50th, and 75th percentiles across all counties, while the lines indicate the full range across counties and the dots indicate the national-level rate

A total of 1,659,045 deaths due to chronic kidney disease were recorded between 1980 and 2014 (477,332 due to diabetes mellitus, 1,056,150 due to hypertension, 122,795 due to glomerulonephritis, and 2768 due to other causes). Chronic kidney disease mortality varied among counties (Fig. 2). Very low mortality rates were observed in central Colorado as well as some counties in southern Florida, California, and Great Plains states. On the other hand, high mortality rates were observed in counties throughout much of the Deep South and a cluster of counties with particularly high rates was observed around the Mississippi river. The lowest estimated mortality rate in 2014 was observed in Summit County, Colorado (4.9 [95% UI: 4.4–5.6] deaths per 100,000 population), while the highest was observed in East Carroll Parish, Louisiana (70.2 [95% UI: 64.4–76.2] deaths per 100,000 population). Nationally, the mortality rate due to CKD was relatively stable between 1980 and 1990 (percent change: − 2.4% [95% UI: − 6.4 to 1.7%]) but increased by 50.1% (95% UI: 43.3–57.1%) between 1990 and 2014. CKD mortality rates increased in most counties between 1980 and 2014 (97.3%; statistically significant in 94.3%). Counties with the largest increases were located primarily in western Oregon, Iowa and Minnesota, southern Illinois, and parts of Texas, Tennessee, Kentucky, and West Virginia. Similar geographic patterns were observed for males and females in 2014 (Additional file 1: eFigs. S3 and S4), although females experienced a larger relative increase in the CKD mortality rate between 1980 and 2014 than men (56.8% [95% UI: 44.8–69.0%] vs. 31.5% [95% UI: 21.1–41.8%]).

### Chronic kidney disease by underlying cause

**Fig. 3:**
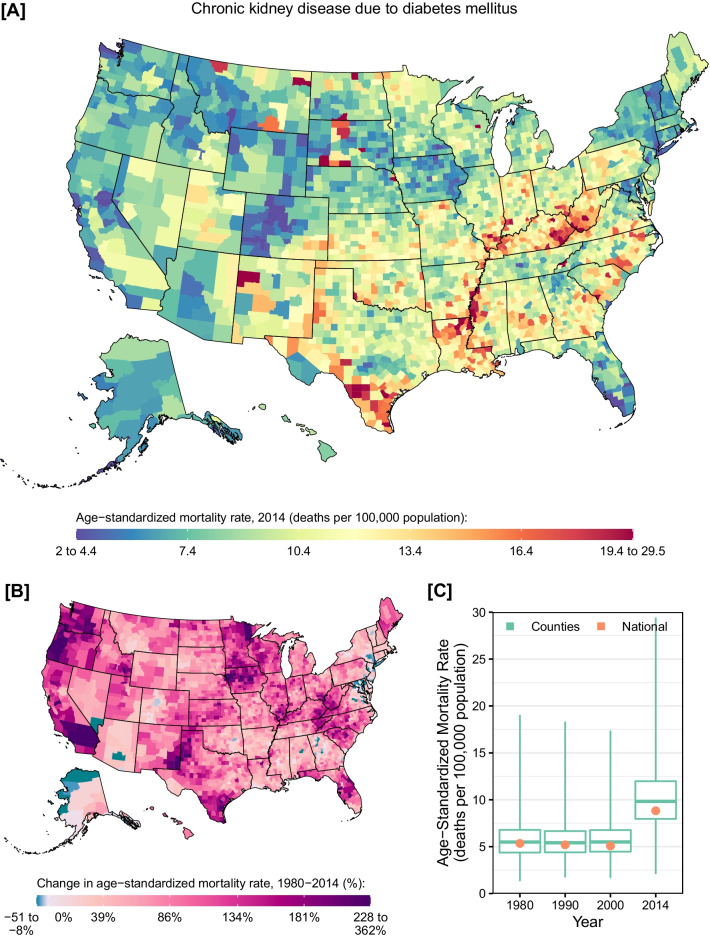
County-level mortality from chronic kidney disease due to diabetes mellitus. **A** Age-standardized mortality rate in 2014; **B** relative change in the age-standardized mortality rate between 1980 and 2014; **C** age-standardized mortality rate in 1980, 1990, 2000, and 2014. In panels **A** and **B**, the color scale is truncated at approximately the 1st and 99th percentiles as indicated by the range given in the color scale. In panel **C**, the boxes indicate the 25th, 50th, and 75th percentiles across all counties, while the lines indicate the full range across counties and the dots indicate the national-level rate

When CKD deaths were examined separately by underlying cause (i.e., diabetes, hypertension, glomerulonephritis, or other factors), several geographic patterns emerged. Counties with very high mortality rates from CKD due to diabetes were found along the Mississippi river, near the border of West Virginia and Kentucky, in southern Texas, in New Mexico, and in North and South Dakota (Fig. 3). Nationally, mortality rates from CKD due to diabetes mellitus declined by 5.0% (95% UI: 1.0–9.1%) between 1980 and 2000, but increased by 73.8% (95% UI: 64.2–88.6%) between 2000 and 2014. Almost all counties experienced an increase in the mortality rate from CKD due to diabetes mellitus between 1980 and 2014 (97.8%; statistically significant in 96.0%). Counties with the largest increases in the mortality rate were located predominantly in west-coast states, Texas, and the northern Midwest. Conversely, counties where mortality declined were located primarily in eastern states and Alaska. Similar geographic patterns were observed for mortality rates among males and females in 2014 (Additional file 1: eFigs. S5 and S6), but the mortality rate was higher overall among males compared to females (10.7 [95% UI: 9.8–12.0] vs. 7.5 [95% UI: 6.8–8.5] deaths per 100,000 population in 2014).

**Fig. 4:**
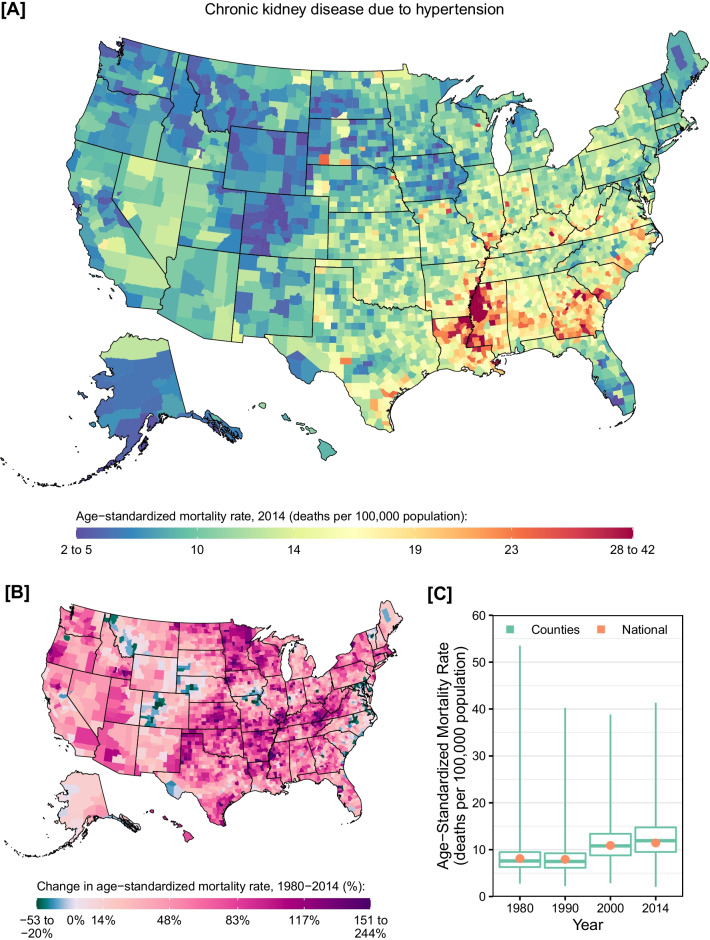
County-level mortality from chronic kidney disease due to hypertension. **A** Age-standardized mortality rate in 2014; **B** relative change in the age-standardized mortality rate between 1980 and 2014; **C** age-standardized mortality rate in 1980, 1990, 2000, and 2014. In panels **A** and **B**, the color scale is truncated at approximately the 1st and 99th percentiles as indicated by the range given in the color scale. In panel **C**, the boxes indicate the 25th, 50th, and 75th percentiles across all counties, while the lines indicate the full range across counties and the dots indicate the national-level rate

Counties with high CKD mortality rates due to hypertension were mainly concentrated in the Southeast with the exception of Florida (Fig. 4). Clusters of counties with particularly high mortality rates were present around the Mississippi river in Mississippi, Louisiana, and Arkansas as well as in Georgia and parts of South Carolina. National mortality rates were relatively stable between 1980 and 1990 (percent change: −2.2% [95% UI: −6.2 to 2.1%]), but increased by 44.0% (95% UI: 34.4–52.5%) between 1990 and 2014. Counties with relatively high rates of increase in the mortality rate between 1980 and 2014 were found primarily in more central states, from Texas in the south, to Minnesota in the north, and West Virginia in the east. Similar geographic and time trends were observed among males and females (Additional file 1: eFigs. S7 and S8), although males experienced a higher mortality rates in 2014 compared to females (13.1 [95% UI: 11.8–14.1] vs. 10.2 [95% UI: 9.3–11.1] deaths per 100,000).

**Fig. 5:**
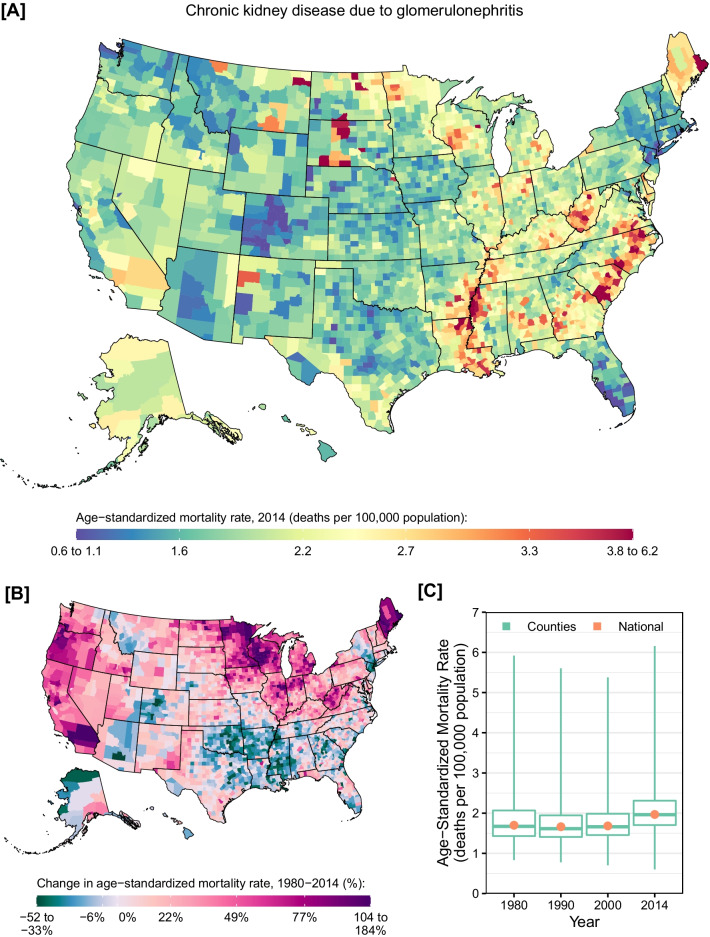
County-level mortality from chronic kidney disease due to glomerulonephritis. **A** Age-standardized mortality rate in 2014; **B** relative change in the age-standardized mortality rate between 1980 and 2014; **C** age-standardized mortality rate in 1980, 1990, 2000, and 2014. In panels **A** and **B**, the color scale is truncated at approximately the 1st and 99th percentiles as indicated by the range given in the color scale. In panel **C**, the boxes indicate the 25th, 50th, and 75th percentiles across all counties, while the lines indicate the full range across counties and the dots indicate the national-level rate

Mortality rates from CKD due to glomerulonephritis varied widely among counties (Fig. 5). Clusters of counties with particularly high mortality rates were observed in the south along the Mississippi river, parts of South and North Carolina, in some counties in North and South Dakota. Nationally, mortality rates were relatively stable from 1980 to 2000 (percent change: −1.0% [95% UI: −5.9 to 4.4%]) but increased by 16.9% (95% UI: 7.8–24.8%) from 2000 to 2014, and counties with particularly large increases were found on the West Coast, in the Midwest, and in Maine. Similar patterns were observed for males and females in 2014 (Additional file 1: eFigs. S9 and S10).

**Fig. 6:**
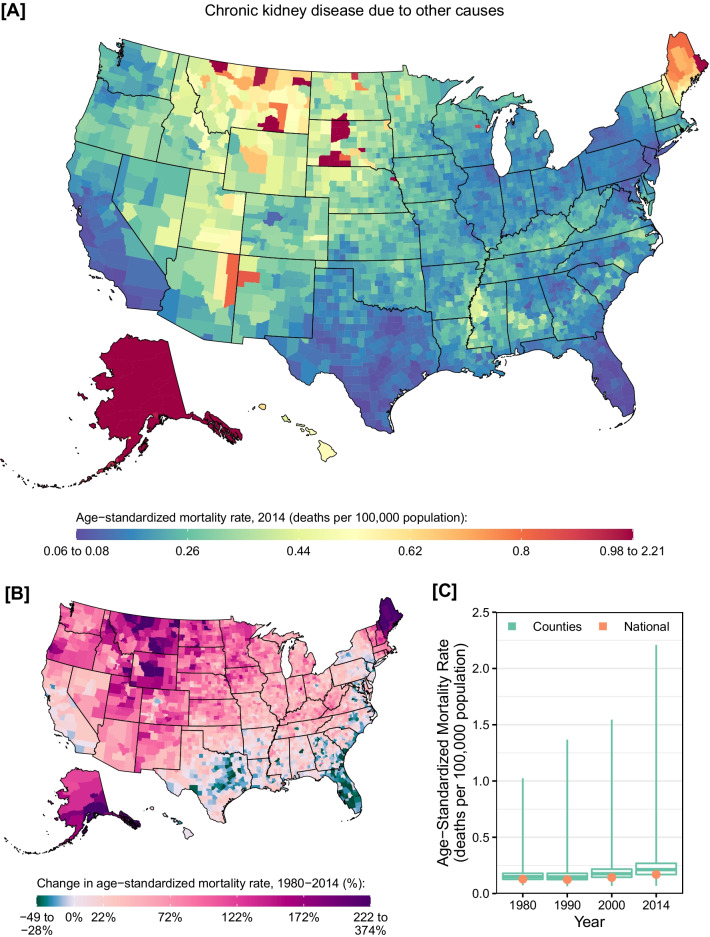
County-level mortality from chronic kidney disease due to other causes. **A** Age-standardized mortality rate in 2014; **B** relative change in the age-standardized mortality rate between 1980 and 2014; **C** age-standardized mortality rate in 1980, 1990, 2000, and 2014. In panels **A** and **B**, the color scale is truncated at approximately the 1st and 99th percentiles as indicated by the range given in the color scale. In panel **C**, the boxes indicate the 25th, 50th, and 75th percentiles across all counties, while the lines indicate the full range across counties and the dots indicate the national-level rate

CKD mortality due other causes had very different geographic patterns compared to CKD mortality overall (Fig. 6). Very high rates were observed in counties in Alaska and parts of Montana, North and South Dakota, and Maine. Nationally, mortality rates increased by 32.7% (95% UI: 22.9–42.9%) between 1980 and 2014, with clusters of high increases in Maine, northern Great Plains states, and Alaska. However, some counties, primarily in southern and eastern states and in California, experienced a decline in mortality over this period. Similar patterns were observed for males and females in 2014 (Additional file 1: eFigs. S11 and S12), although mortality rates were higher overall among males compared to females in 2014 (0.21 [95% UI: 0.16–0.28] vs. 0.14 [95% UI: 0.11–0.19] deaths per 100,000 population).

## Discussion

This study revealed large differences among US counties in mortality rates from diabetes and CKD. The findings showed little improvement in age-standardized mortality rates from diabetes and significant increases in age-standardized mortality rates due to CKD between 1980 and 2014. Diabetes mortality actually increased substantially from 1980 to 2000 with a downward trend thereafter. On the other hand, CKD mortality increased sharply in recent decades. These findings will assist in examining the root causes of these trends and disparities among US counties. Moreover, they will provide valuable insights into developing and implementing programs and policies to reduce the burden and disparities of diabetes and CKD mortality.

This study showed that diabetes mortality has declined at the national level from 2000 to 2014 at a time when the prevalence of diabetes was increasing [2]. This is the result of a decline in the case fatality rate due to better treatment and management of diabetes. However, there are huge variations in the rate of decline by counties indicating the need for better access to medical care throughout the country [28].

These results showed a rapid increase in CKD mortality from 1990 to 2014 in the USA. Several studies have reported little improvement in CKD prevalence in the USA from 1990 to 2015 [29]. This finding is a surprising since there are known effective interventions such ACE inhibitors to prevent CKD progression [30]. This finding has several implications. CKD is a disease that requires extensive medical care and resources such as dialysis and others [8]. Indeed, this rise will put a lot of strains on the medical system in the coming years.

CKD mortality due to diabetes and hypertension increased in recent years, underscoring the need for better treatment and management of blood pressure and diabetes. Both diabetes and blood pressure diagnosis and control are not optimal in the USA. Several studies have shown that counties have large variations in diagnoses and control of diabetes and blood pressure [28, 31]. Early detection through screening of high-risk individuals is crucial to control blood pressure and diabetes and reduce diabetes and CKD burden and mortality [32]. Early diagnosis will facilitate treatment and behavioral changes and will increase survival. There is a need for more aggressive programs to control blood pressure and diabetes that include medical and preventive care approaches.

Access to and quality of medical care have a major impact on mortality from diabetes and CKD [33]. Several studies have shown that timely diagnosis of diabetes and CKD and proper treatment reduce the complications and improve the outcome [32, 34]. Unfortunately, not all US residents have equal access to quality medical care. Both diabetes and CKD require that the patient adhere to long-term management of the condition [35]. It is possible that proper management of these conditions vary by county and have led to these disparities. However, for many physicians in the USA, especially in poor and rural areas where the patient load is heavy, they have little or no time for patient counselling. In addition, the current health system financing does not reimburse health facilities for time spent on patient education.

Several studies have shown that obesity has rapidly increased in the USA during the time period of this study [36, 37]. In fact, some studies have called obesity an epidemic as it has impacted all geographic areas and demographic groups [6]. Recent reports showed a slight improvements in physical activity, but many individuals in the USA do not meet the recommended levels of physical activity [36]. There is a need for programs to improve physical activity in the USA to reduce the burden of diabetes as well as many other conditions.

Diet is a major risk factor for type 2 diabetes and for CKD [5]. For example, low intake of whole grains and the consumption of processed food and red meats are known risk factors for type 2 diabetes [9]. Also, diet high in salt consumption is associated with an increased blood pressure [38]. The 2019 Global Burden of Disease showed that poor diet is a major cause of type 2 diabetes [29]. In the USA, diet has not improved much during the study period [39]. Moreover, there is only limited information on dietary habits at the local level. The only available source for county dietary intake is 6 questions on fruits and vegetables from the behavioral risk factor surveillance system [40].

The Diabetes Prevention Program and Outcomes Study shows that type 2 diabetes is largely preventable through healthy lifestyle [41]. The study showed that lifestyle interventions were also cost-effective. However, the long-term effects of such a program have not been well documented. It is time to invest in prevention activities in communities in order to improve behaviors. Perhaps, sponsoring innovations is a mean to find local solutions since counties and communities need financial and technical support to solve these challenges.

Diabetes and CKD are associated with poor quality of life [42]. Several studies have shown that diabetes patients have more days of poor mental and physical health [42, 43]. They are more likely to miss work and be less productive [44]. Therefore, social support initiatives for patients and their care givers are important for improving quality of life.

Our study showed high levels of diabetes and CKD mortality in areas in the South East, especially around the Mississippi river. Previous research has identified differences in socioeconomic status, differences in the prevalence of behavioral and metabolic risk factors, and differences in access to and quality of health care as potential drivers of differences in life expectancy among counties, with risk factors explaining the largest proportion of the differences in life expectancy [45]. Unfortunately, areas with high diabetes and CKD mortality in our manuscript have high levels of obesity, low levels of physical activity, high levels of smoking, and poor diet. Addressing these risk factors would be the best strategy to reduce the burden of many chronic conditions.

This study has a number of important limitations. First, the data sources underlying this analysis, i.e., deaths, population, and covariates, are each subject to error. Second, the garbage code redistribution methods used for this analysis have not been validated due to a lack of appropriate gold standard data. Third, uncertainty due to garbage code redistribution methods has not been quantified and included in the uncertainty intervals reported for this analysis. Fourth, the small area estimation models smooth mortality rates over space, time, and age group which may attenuate unusually low or high mortality rates, leading to an underestimation of geographic variation.

## Conclusions

This study found large inequalities among counties in diabetes and CKD mortality. The findings provide insights into the root causes of this variation. Risk factors, access to medical care, and quality of medical care must improve in many parts of the country to reduce the burden of these diseases. As the US population grows and ages, and as medical costs continue to rise, renewed focus on prevention efforts are urgently required.

## Supplementary Information

**Additional file 1:** Supplemental Online Content.

## Abbreviations

BRFSS: Behavioral risk factor surveillance systems
CKD: Chronic kidney disease
GBD: Global burden of disease
ICD: International classification of diseases
NCHS: National Center for Health Statistics
YLLs: Years of life lost
