# Supplementary material for: Trends and patterns of disparities in diabetes and chronic kidney disease mortality among US counties, 1980–2014
Source: Popul Health Metr. 2022 Feb 22;20:9. doi: 10.1186/s12963-022-00285-4 (PMC8862531; doi:10.1186/s12963-022-00285-4)
Supplement: Supplementary file 1 — Additional file 1: Supplemental Online Content. [file 12963_2022_285_MOESM1_ESM.pdf]

Supplemental Online Content: Trends and patterns of disparities in diabetes and chronic kidney disease mortality among US counties, 1980-2014.

eTables

eTable 1: Data sources used for covariates.

eTable 2: Counties combined to ensure historically stable units of analysis.

eTable 3: GBD cause list and associated ICD9 and ICD10 codes.

eFigures

eFigure 1: County-level mortality among males from diabetes mellitus.

eFigure 2: County-level mortality among females from diabetes mellitus.

eFigure 3: County-level mortality among males from chronic kidney disease.

eFigure 4: County-level mortality among females from chronic kidney disease.

eFigure 5: County-level mortality among males from chronic kidney disease due to diabetes mellitus.

eFigure 6: County-level mortality among females from chronic kidney disease due to diabetes mellitus.

eFigure 7: County-level mortality among males from chronic kidney disease due to hypertension.

eFigure 8: County-level mortality among females from chronic kidney disease due to hypertension.

eFigure 9: County-level mortality among males from chronic kidney disease due to glomerulonephritis.

eFigure 10: County-level mortality among females from chronic kidney disease due to glomerulonephritis.

eFigure 11: County-level mortality among males from chronic kidney disease due to other causes.

eFigure 12: County-level mortality among females from chronic kidney disease due to other causes.

eTable 1: Data sources used for covariates.

Data Sources	Data Processing
Percent of the population age 25 and older who have completed high school	
1980 census [1]; 1990 census [2]; 2000 census [3]; 2009-2014 ACS [4-9]	Linear interpolation was used fill in intermediate years between data sources. The rate of change calculated between 2007 and 2012 was applied to fill in estimates for 2013 and 2014.
Percent of the population who are Hispanic	
1980 census [10]; 1990-2014 NCHS Bridged Race Files [11-13]	Linear interpolation was used to fill in intermediate years between data sources.
Percent of the population who are Black and some other race	
1980-1989 Census Bureau Intercensal County Estimates by Age, Sex, and Race [14]; 1990-2014 NCHS Bridged Race Files [11-13]	Linear interpolation was used to fill in intermediate years between data sources.
Percent of land area in a Native American reservation	
2013 Cartographic Boundary File, State-County for United States [15]; AIANNH Areas National Shapefile [16]	Geographic boundaries of AIANNH Areas were intersected with county boundaries using ArcGIS. The area of the intersection and the area of the county were calculated using an Albers Equal Area Conic projection. The proportion of the land area that is in a reservation was generated by dividing the area of the reservation by the total area in each county.
Household Median Income	
1980 census [17]; 1989, 1993, 1995-2014 Small Area Income and Poverty Estimates [18]; 1980-2014 Bureau of Labor Statistics, Consumer Price Index [19]	Data were adjusted for inflation using the consumer price index, and linear interpolation was used to generate values between observed data points. Income was then log-transformed.
Population Density	
1980-1989 Census Bureau Intercensal County Estimates by Age, Sex, and Race [14]; 1990-2014 NCHS Bridged Race Files [11-13]; 2013 Cartographic Boundary File, State-County for United States [15]	The area of each county was calculated using an Albers Equal Area Conic projection. The total population of each county was divided by the total area of the county, and was then log-transformed.

[1] Missouri Census Data Center. 1980 Census Summary Tape File 3, Table NT48A. MCDC Data Archive (Uexplore/Dexter). http://mcdc2.missouri.edu/applications/uexplore.shtml. Accessed April 22, 2013.

[2] Minnesota Population Center. 1990 Census Summary Tape File 3, Table P057. National Historical Geographic Information System: Version 2.0. Minneapolis, MN: University of Minnesota 2011. http://www.nhgis.org. Accessed July 18, 2013.

[3] US Census Bureau. 2000 Census Summary Tape File 3, Table DP2. http://factfinder2.census.gov. Accessed April 18, 2013.

[4] US Census Bureau. 2009 American Community Survey 5-Year Estimates, Table S1501. http://factfinder2.census.gov. Accessed April 17, 2013.

[5] US Census Bureau. 2010 American Community Survey 5-Year Estimates, Table S1501. http://factfinder2.census.gov. Accessed April 17, 2013.

[6] US Census Bureau. 2011 American Community Survey 5-Year Estimates, Table S1501. http://factfinder2.census.gov. Accessed April 17, 2013.

[7] US Census Bureau. 2012 American Community Survey 5-Year Estimates, Table S1501. http://factfinder2.census.gov. Accessed December 18, 2013.

[8] US Census Bureau. American Community Survey, 2013 American Community Survey 5-Year Estimates, Table S1501. http://factfinder2.census.gov. Accessed December 23, 2014.

[9] US Census Bureau. American Community Survey, 2014 American Community Survey 5-Year Estimates, Table S1501. http://factfinder2.census.gov. Accessed December 8, 2015.

[10] Minnesota Population Center. 1980 Census Summary Tape File 1, Table NT8. National Historical Geographic Information System: Version 2.0. Minneapolis, MN: University of Minnesota 2011. http://www.nhgis.org. Accessed January 13, 2016.

[11] National Center for Health Statistics. United States Bridged-Race Intercensal Population Estimates 1990-1999. http://www.cdc.gov/nchs/nvss/bridged_race.htm. Accessed November 21, 2011.

[12] National Center for Health Statistics. United States Bridged-Race Intercensal Population Estimates 2000-2009. http://www.cdc.gov/nchs/nvss/bridged_race.htm. Accessed October 30, 2012.

[13] National Center for Health Statistics. United States Vintage 2014 Bridged-Race Postcensal Population Estimates 2010-2014. http://www.cdc.gov/nchs/nvss/bridged_race.htm. Accessed December 18, 2015.

[14] US Census Bureau. Intercensal County Estimates by Age, Sex, Race: 1980-1989. http://www.census.gov/popest/data/counties/asrh/1980s/PE-02.html. Accessed January 8, 2015.

[15] US Census Bureau. TIGER/Line Shapefile, 2013 Cartographic Boundary File, State-County for United States, 1:20,000,000. https://catalog.data.gov/dataset/2013-cartographic-boundary-file-state-county-for-united-states-1-20000000. Accessed February 2, 2015.

[16] US Census Bureau. TIGER/Line Shapefile, 2012, Series Information File for the Nation, Current American Indian/Alaska Native/Native Hawaiian Areas (AIANNH) National Shapefile. http://catalog.data.gov/dataset/tiger-line-shapefile-2012-series-information-file-for-the-nation-current-american-indian-alaska. Accessed February 10, 2015.

[17] Minnesota Population Center. Summary Tape File 3, Table NT69. National Historical Geographic Information System: Version 2.0. Minneapolis, MN: University of Minnesota 2011. http://www.nhgis.org. Accessed November 12, 2015.

[18] US Census Bureau. Small Area Income and Poverty Estimates. https://www.census.gov/did/www/saipe/data/statecounty/data/index.html. Accessed December 28, 2015.

[19] US Bureau of Labor Statistics. Consumer Price Index: All Urban Consumers History, All Items 1913-2015. http://www.bls.gov/data/. Accessed March 25, 2015.

eTable 2: Counties combined to ensure historically stable units of analysis.

State	Group	Areas
Alaska	1	Kusilvak Census Area (2158), Wade Hampton Census Area (2270)*
	2	Kobuk Census Area (2140)*, Northwest Arctic Borough (2188)
	3	Aleutian Islands Census Area (2010)*, Aleutians East Borough (2013), Aleutians West Census Area (2016)
	4	Dillingham Census Area (2070), Lake and Peninsula Borough (2164)
	5	Denali Borough (2068), Yukon-Koyukuk Census Area (2290)
	6	Hoonah-Angoon Census Area (2105), Skagway Municipality (2230), Skagway-Yakutat-Angoon Census Area (2231)*, Skagway-Hoonah-Angoon Census Area (2232)*, Yakutat City and Borough (2282)
	7	Ketchikan Gateway Borough (2130), Petersburg Borough (2195), Prince of Wales-Hyder Census Area (2198), Prince of Wales-Outer Ketchikan Census Area (2201)*, Wrangell City and Borough (2275), Wrangell-Petersburg Census Area (2280)*
Arizona	1	La Paz County (4012), Yuma County (4027)
Colorado	1	Adams County (8001), Arapahoe County (8005), Boulder County (8013), Broomfield County (8014), Denver County (8031), Jefferson County (8059), Weld County (8123)
Florida	1	Dade County (12025)*, Miami-Dade County (12086)
Hawaii	1	Kalawao County (15005), Maui County (15009)
Maryland	1	Montgomery County (24031), Prince George's County (24033)
Montana	1	Park County (30067), Yellowstone National Park (30113)*
New Mexico	1	Cibola County (35006), Valencia County (35061)
South Dakota	1	Oglala Lakota County (46102), Shannon County (46113)*
	2	Jackson County (46071), Washabaugh County (46131)*
Virginia	1	Fairfax County (51059), Fairfax City (51600)
	2	Rockingham County (51165), Harrisonburg City (51660)
	3	James City County (51095), Williamsburg City (51830)
	4	Prince William County (51153), Manassas City (51683), Manassas Park City (51685)
	5	Rockbridge County (51163), Buena Vista City (51530)
	6	Spotsylvania County (51177), Fredericksburg City (51630)
	7	Augusta County (51015), Staunton City (51790), Waynesboro City (51820)
	8	Pittsylvania County (51143), Danville City (51590)
	9	Greensville County (51081), Emporia City (51595)
	10	Albemarle County (51003), Charlottesville City (51540)
	11	Bedford County (51019), Bedford City (51515)*
	12	Halifax County (51083), South Boston City (51780)*
	13	Southampton County (51175), Franklin City (51620)
	14	Alleghany County (51005), Clifton Forge City (51560)*
	15	York County (51199), Newport News City (51700)

*County no longer exists due to boundary or name change.

eTable 3: GBD cause list and associated ICD9 and ICD10 codes.

Cause	Level	ICD9	ICD10
Communicable, maternal, neonatal, and nutritional diseases	1	001-001.9, 002.0-030.9, 032-034.9, 036-036.40, 036.5, 036.8-037.9, 039-039.4, 039.8-040, 040.1-041.09, 042-066.9, 070-070.21, 070.3-070.31, 070.4-070.43, 070.49-070.53, 070.59-074.1, 074.20, 074.3-075.9, 078.4-078.7, 079-079.7, 080-083.9, 084.0-084.5, 084.7-084.9, 085.0, 086-088.9, 090-101.6, 104-104.9, 120-124.9, 125.4-125.9, 127-127.1, 128-129.0, 136-136.29, 137-139.0, 244.2, 260-263.9, 265-269.9, 280.1-280.8, 281.0-281.9, 320.0-320.89, 321-323.9, 381-383.9, 390-390.9, 392, 392.9, 461-461.9, 464.0, 464.01, 464.11-464.2, 464.21, 464.31-464.4, 464.8-464.9, 466-469, 470.0, 475-475.9, 476.9, 480-482.89, 483.0-483.9, 484.0-484.7, 487-489, 613-614.9, 630-636.92, 638-638.92, 640-679.14, 716.0-716.09, 730.4-730.6, 760-760.64, 760.8-768, 768.2-770, 770.1-775, 775.4-779.34, 779.6-779.89, 787.91	A00-A00.9, A01.0-A14, A15-A28.9, A30-A30.9, A32-A39.4, A39.8-A39.9, A48.1-A48.2, A48.4-A48.52, A49.1, A50-A58, A60-A60.9, A63-A63.8, A65-A65.0, A68-A70, A74, A74.8-A75.9, A77-A96.9, A98-A98.8, B00-B06.9, B10-B10.89, B15-B17.9, B19-B27.99, B29.4, B33-B33.1, B33.3-B33.8, B47-B48.8, B50-B53.8, B55.0, B56-B57.5, B60-B60.8, B63, B65-B67.99, B69-B72.0, B74.3-B75, B77-B77.9, B83-B83.8, B90-B92, B94.1-B94.2, B95-B95.5, D50.1-D50.8, D51-D52.0, D52.8-D53.9, D64.3, D86.81, E00-E02, E40-E46.9, E51-E61.9, E63-E64.0, E64.2-E64.9, F07.1, G00.0-G00.8, G03-G03.8, G04-G05.8, G14-G14.6, H70-H70.93, I00, I02, I02.9, I98.0-I98.1, J01-J01.91, J02.0, J03.0-J03.01, J04.0, J05-J05.0, J05.11, J09-J15.8, J16-J16.9, J20-J21.9, J36-J36.0, K67.0-K67.8, K74.7-K74.8, K93.0, M03.1, M12.1-M12.19, M49.0-M49.1, M73.0-M73.1, M89.6-M89.69, N70-N71.9, N73-N74.8, N96, N98-N98.9, O00-O07.9, O09-O16.9, O20-O26.93, O28-O36.93, O40-O48.1, O60-O77.9, O80-O92.79, O96-P04.2, P04.5-P05.9, P07-P15.9, P19-P22.9, P23.0-P23.4, P24-P29.9, P35-P37.2, P37.5-P39.9, P50-P61.9, P70, P70.3-P72.9, P74-P78.9, P80-P81.9, P83-P84, P90-P94.9, P96, P96.3-P96.4, P96.8-P96.89, R19.7
HIV/AIDS and tuberculosis	2	010-019.9, 042-044.9, 137-137.9, 138.0-138.9, 730.4-730.6	A10-A14, A15-A19.9, B20-B24.9, B90-B90.9, K67.3, K93.0, M49.0, P37.0
Tuberculosis	3-4	010-019.9, 137-137.9, 138.0-138.9, 730.4-730.6	A10-A14, A15-A19.9, B90-B90.9, K67.3, K93.0, M49.0, P37.0
HIV/AIDS	3	042-044.9	B20-B24.9
HIV/AIDS - Tuberculosis	4		B20.0
HIV/AIDS resulting in other diseases	4	042.0-042.9, 043.0-043.9, 044.1-044.9	B20.1-B23.9, B24.0
Diarrhea, lower respiratory, and other common infectious diseases	2	001-001.9, 002.0-009.9, 032-033.9, 036-036.40, 036.5, 036.8-037.9, 047-049.9, 052-053.9, 055-055.9, 062-064.9, 073.0-073.6, 139.0, 320.0-320.89, 321-323, 323.1, 323.4-323.9, 381-383.9, 461-461.9, 464.0, 464.01, 464.11-464.2, 464.21, 464.31-464.4, 464.8-464.9, 466-469, 470.0, 475-475.9, 476.9, 480-482.89, 483.0-483.9, 484.0-484.4, 484.6-484.7, 487-489, 771.3, 787.91	A00-A00.9, A01.0-A09.9, A33-A37.91, A39-A39.4, A39.8-A39.9, A48.1, A70, A83-A87.9, B01-B02.9, B05-B05.9, B94.1, D86.81, F07.1, G00.0-G00.8, G03-G03.8, G04-G05.8, H70-H70.93, J01-J01.91, J04.0, J05-J05.0, J05.11, J09-J15.8, J16-J16.9, J20-J21.9, J36-J36.0, P23.0-P23.4, P35.8, R19.7
Diarrheal diseases	3-4	001-001.9, 003-006.9, 007.4-007.8, 008.01-008.02, 008.04, 008.2-009.9, 787.91	A00-A00.9, A02-A04.1, A04.3, A04.5-A07, A07.2-A07.4, A08-A09.9, R19.7
Intestinal infectious diseases	3	002.0-002.9, 007-007.3, 007.9-008.00, 008.03, 008.09-008.1	A01.0-A01.4, A04.2, A04.4, A07.0-A07.1, A07.8-A07.9
Typhoid fever	4	2	A01.0-A01.09
Paratyphoid fever	4	002.1-002.9	A01.1-A01.4
Other intestinal infectious diseases	4	007-007.3, 007.9-008.00, 008.03, 008.09-008.1	A04.2, A04.4, A07.0-A07.1, A07.8-A07.9
Lower respiratory infections	3-4	073.0-073.6, 466-469, 470.0, 480-482.89, 483.0-483.9, 484.1-484.2, 484.6-484.7, 487-489	A48.1, A70, J09-J15.8, J16-J16.9, J20-J21.9, P23.0-P23.4
Upper respiratory infections	3-4	461-461.9, 464.0, 464.01, 464.11-464.2, 464.21, 464.31-464.4, 464.8-464.9, 475-475.9, 476.9	J01-J01.91, J04.0, J05-J05.0, J05.11, J36-J36.0
Otitis media	3-4	381-383.9	H70-H70.93
Meningitis	3	036-036.40, 036.5, 036.8-036.9, 047-049.9, 320.0-320.89, 321-322.9	A39-A39.4, A39.8-A39.9, A87-A87.9, D86.81, G00.0-G00.8, G03-G03.8

eTable 3: GBD cause list and associated ICD9 and ICD10 codes (continued).

Cause	Level	ICD9	ICD10
Pneumococcal meningitis	4	320.1	G00.1
H influenzae type B meningitis	4	320	G00.0
Meningococcal meningitis	4	036-036.40, 036.5, 036.8-036.9	A39-A39.4, A39.8-A39.9
Other meningitis	4	047-049.9, 320.2-320.89, 321-322.9	A87-A87.9, D86.81, G00.2-G00.8, G03-G03.8
Encephalitis	3-4	062-064.9, 139.0, 323, 323.4-323.9	A83-A86.4, B94.1, F07.1, G04-G05.8
Diphtheria	3-4	032-032.9	A36-A36.9
Whooping cough	3-4	033-033.9, 484.3-484.4	A37-A37.91
Tetanus	3-4	037-037.9, 771.3	A33-A35.0
Measles	3-4	055-055.9, 323.1, 484.0	B05-B05.9
Varicella and herpes zoster	3-4	052-053.9	B01-B02.9, P35.8
Neglected tropical diseases and malaria	2	030-030.9, 060-061.8, 065-066.9, 071-071.9, 080, 080.2-083.9, 084.0-084.5, 084.7-084.9, 085.0, 086-088.9, 120-124.9, 125.4-125.9, 127-127.1, 128-129.0	A30-A30.9, A68-A68.9, A69.2-A69.9, A75-A75.9, A77-A79.9, A82-A82.9, A90-A96.9, A98-A98.8, B33.0-B33.1, B50-B53.8, B55.0, B56-B57.5, B60-B60.8, B65-B67.99, B69-B72.0, B74.3-B75, B77-B77.9, B83-B83.8, B92, P37.1
Malaria	3-4	084.0-084.5, 084.7-084.9	B50-B53.8
Chagas disease	3-4	086-086.2, 086.9	B57-B57.5
Leishmaniasis	3	85	B55.0
Visceral leishmaniasis	4	85	B55.0
African trypanosomiasis	3-4	086.3-086.5	B56-B56.9
Schistosomiasis	3-4	120-120.9	B65-B65.9
Cysticercosis	3-4	123.1	B69-B69.9
Cystic echinococcosis	3-4	122-122.4, 122.8-122.9	B67-B67.4, B67.8-B67.99
Dengue	3-4	061-061.8	A90-A91.9
Yellow fever	3-4	060-060.9	A95-A95.9
Rabies	3-4	071-071.9	A82-A82.9
Intestinal nematode infections	3	127	B77-B77.9
Ascariasis	4	127	B77-B77.9
Ebola	3-4		A98.4
Other neglected tropical diseases	3-4	065-066.9, 080, 080.2-083.9, 087-088.9, 122.5-122.7, 123-123.0, 123.2-124.9, 125.4-125.9, 127, 127.1, 128-129.0	A68-A68.9, A69.2-A69.9, A75-A75.9, A77-A79.9, A92-A94.0, A96-A96.9, A98-A98.3, A98.5-A98.8, B33.0-B33.1, B60-B60.8, B67.5-B67.7, B70-B72.0, B74.3-B75, B83-B83.8, P37.1
Maternal disorders	2	630-636.92, 638-638.92, 640-679.14	N96, N98-N98.9, O00-O07.9, O09-O16.9, O20-O26.93, O28-O36.93, O40-O48.1, O60-O77.9, O80-O92.79, O96-O99.91
Maternal hemorrhage	3-4	640-641.93, 661-661.93, 665, 666-666.9	O20-O20.9, O43.2-O43.239, O44-O46.93, O62-O62.9, O67-O67.9, O70, O72-O72.3
Maternal sepsis and other maternal infections	3-4	659.3-659.33, 670-670.9	O23-O23.93, O85-O86.89, O91-O91.23
Maternal hypertensive disorders	3-4	642-642.94	O10-O16.9
Maternal obstructed labor and uterine rupture	3-4	652-653.93, 660-660.93, 665.0-665.34	O32-O33.9, O64-O66.9, O71-O71.9
Maternal abortion, miscarriage, and ectopic pregnancy	3-4	630-636.92, 638-638.92, 646.3-646.33	N96, O00-O07.9
Indirect maternal deaths	3-4	646-646.24, 646.4-649.9, 674-674.94	O24-O25.3, O98-O99.91
Late maternal deaths	3-4		O96-O97.9
Maternal deaths aggravated by HIV/AIDS	3-4		
Other maternal disorders	3-4	643-645.23, 650-651.93, 654-659.23, 659.4-659.93, 662-664.94, 665.4-665.94, 667-669.94, 671-673.9, 675-679.14	N98-N98.9, O09-O09.93, O21-O22.93, O26-O26.93, O28-O31.8, O34-O36.93, O40-O43.199, O43.8-O43.93, O47-O48.1, O60-O61.9, O63-O63.9, O68-O69.9, O70.0-O70.9, O73-O77.9, O80-O84.9, O87-O90.9, O92-O92.79
Neonatal disorders	2	760-760.64, 760.8-768, 768.2-770, 770.1-771, 771.4-775, 775.4-779.34, 779.6-779.89	P00-P04.2, P04.5-P05.9, P07-P15.9, P19-P22.9, P24-P29.9, P36-P36.9, P38-P39.9, P50-P61.9, P70, P70.3-P72.9, P74-P78.9, P80-P81.9, P83-P84, P90-P94.9, P96, P96.3-P96.4, P96.8-P96.89
Neonatal preterm birth complications	3-4	761.0-761.1, 765-765.9, 769-769.9, 770.2-770.9, 776.6, 777.5-777.6	P01.0-P01.1, P07-P07.39, P22-P22.9, P25-P28.9, P61.2, P77-P77.9

eTable 3: GBD cause list and associated ICD9 and ICD10 codes (continued).

Cause	Level	ICD9	ICD10
Neonatal encephalopathy due to birth asphyxia and trauma	3-4	761.7-763.9, 767-768, 768.2-768.9, 770.1-770.18, 772.1-772.9, 779.0-779.2	P01.7, P02-P03.9, P10-P15.9, P20-P21.9, P24-P24.9, P90-P91.9
Neonatal sepsis and other neonatal infections	3-4	771.4-771.9	P36-P36.9, P38-P39.9
Hemolytic disease and other neonatal jaundice	3-4	773-774.9	P55-P59.9
Other neonatal disorders	3-4	760-760.64, 760.8-761, 761.2-761.6, 764-764.99, 766-766.9, 770, 771, 772-772.0, 775, 775.4-776.5, 776.7-777.4, 777.7-779, 779.3-779.34, 779.6-779.89	P00-P01, P01.2-P01.6, P01.8-P01.9, P04-P04.2, P04.5-P05.9, P08-P09, P19-P19.9, P29-P29.9, P50-P54.9, P60-P61.1, P61.3-P61.9, P70, P70.3-P72.9, P74-P76.9, P78-P78.9, P80-P81.9, P83-P84, P92-P94.9, P96, P96.3-P96.4, P96.8-P96.89
Nutritional deficiencies	2	244.2, 260-263.9, 265-269.9, 280.1-280.8, 281.0-281.9, 716.0-716.09	D50.1-D50.8, D51-D52.0, D52.8-D53.9, D64.3, E00-E02, E40-E46.9, E51-E61.9, E63-E64.0, E64.2-E64.9, M12.1-M12.19
Protein-energy malnutrition	3-4	260-263.9	E40-E46.9, E64.0
Iodine deficiency	3-4	244.2	E00-E02
Iron-deficiency anemia	3-4	280.1-280.8	D50.1-D50.8, D64.3
Other nutritional deficiencies	3-4	265-269.9, 281.0-281.9, 716.0-716.09	D51-D52.0, D52.8-D53.9, E51-E61.9, E63-E64, E64.2-E64.3, M12.1-M12.19
Other communicable, maternal, neonatal, and nutritional diseases	2	020-029, 034-034.9, 039-039.4, 039.8-040, 040.1-041.09, 045-046.9, 050-051.9, 054-054.9, 056-059.9, 070-070.21, 070.3-070.31, 070.4-070.43, 070.49-070.53, 070.59-070.9, 072-073, 073.7-074.1, 074.20, 074.3-075.9, 078.4-078.7, 079-079.7, 080.0, 090-101.6, 104-104.9, 136-136.29, 138, 139, 323.0-323.02, 323.2-323.3, 390-390.9, 392, 392.9, 484.5, 613-614.9, 771.0-771.2	A20-A28.9, A32-A32.9, A38-A38.9, A48.2, A48.4-A48.52, A49.1, A50-A58, A60-A60.9, A63-A63.8, A65-A65.0, A69-A69.1, A74, A74.8-A74.9, A80-A81.9, A88-A89.9, B00-B00.9, B03-B04, B06-B06.9, B10-B10.89, B15-B17.9, B19-B19.9, B25-B27.99, B29.4, B33, B33.3-B33.8, B47-B48.8, B63, B91, B94.2, B95-B95.5, G14-G14.6, I00, I02, I02.9, I98.0-I98.1, J02.0, J03.0-J03.01, K67.0-K67.2, K67.8, K74.7-K74.8, M03.1, M49.1, M73.0-M73.1, M89.6-M89.69, N70-N71.9, N73-N74.8, P35-P35.3, P35.9, P37, P37.2, P37.5-P37.9
Sexually transmitted diseases excluding HIV	3	054.1, 090-099.9, 613-614.9	A50-A58, A60-A60.9, A63-A63.8, B63, I98.0, K67.0-K67.2, M03.1, M73.0-M73.1, N70-N71.9, N73-N74.8
Syphilis	4	090-097.9	A50-A53.9, I98.0, K67.2, M03.1, M73.1
Chlamydial infection	4	099, 099.1-099.6	A55-A56.8, K67.0
Gonococcal infection	4	098-098.9	A54-A54.9, K67.1, M73.0
Other sexually transmitted diseases	4	099.0, 099.8-099.9	A57-A58, A63-A63.8
Hepatitis	3-4	070-070.21, 070.3-070.31, 070.4-070.43, 070.49-070.53, 070.59-070.9	B15-B17.9, B19-B19.9, B94.2, P35.3
Other infectious diseases	3-4	020-029, 034-034.9, 039-039.4, 039.8-040, 040.1-041.09, 045-046.9, 050-051.9, 054-054.0, 054.10-054.9, 056-059.9, 072-073, 073.7-074.1, 074.20, 074.3-075.9, 078.4-078.7, 079-079.7, 080.0, 100-101.6, 104-104.9, 136-136.29, 138, 139, 323.0-323.02, 323.2-323.3, 390-390.9, 392, 392.9, 484.5, 771.0-771.2	A20-A28.9, A32-A32.9, A38-A38.9, A48.2, A48.4-A48.52, A49.1, A65-A65.0, A69-A69.1, A74, A74.8-A74.9, A80-A81.9, A88-A89.9, B00-B00.9, B03-B04, B06-B06.9, B10-B10.89, B25-B27.99, B29.4, B33, B33.3-B33.8, B47-B48.8, B91, B95-B95.5, G14-G14.6, I00, I02, I02.9, I98.1, J02.0, J03.0-J03.01, K67.8, K74.7-K74.8, M49.1, M89.6-M89.69, P35-P35.2, P35.9, P37, P37.2, P37.5-P37.9
Non-communicable diseases	1	035-035.9, 036.41-036.43, 036.6, 070.22-070.23, 070.32-070.33, 070.44, 070.54, 074.2, 074.21-074.23, 102-103.9, 133-133.6, 135-135.9, 136.6, 140-148.9	A39.5-A39.53, A46-A46.0, A66-A67.9, B18-B18.9, B33.2-B33.24, B86, C0-C13.9, C15-C25.9, C3-C34.92, C37-C38.8, C4-C41.9, C43-C45.9

eTable 3: GBD cause list and associated ICD9 and ICD10 codes (continued).

Cause	Level	ICD9	ICD10
Non-communicable diseases (continued)	1	150-158.9, 160-164.9, 170-175.9, 180-183.8, 184.0-184.4, 184.8, 185-186.9, 187.1-187.8, 188-188.9, 189.0-189.8, 190-194.8, 200-208.92, 209.0-209.17, 209.21-209.27, 209.31-209.57, 209.61, 209.63-209.67, 210.0-210.9, 211.0-211.8, 212.0-212.8, 213-213.9, 217-220.9, 221.0-221.8, 222.0-222.8, 223.0-223.89, 224-228.9, 229.0, 229.8, 230.1-230.8, 231.0-231.2, 232-232.9, 233.0-233.2, 233.31-233.32, 233.4-233.5, 233.7, 234.0-234.8, 235.0, 235.4, 235.6-235.8, 236.0-236.2, 236.4-236.5, 236.7, 236.91-237.3, 237.5-237.9, 238.0-238.5, 239.2-239.4, 239.6, 240-243.9, 244.0-244.1, 244.3-244.8, 245-246.9, 250-259.9, 270-273.9, 275-276, 277-277.2, 277.4-277.9, 278.0-278.8, 282-284.9, 286-286.5, 286.7-289.7, 290-292.9, 294.1-295.95, 303-303.93, 304.0-304.83, 305-305.93, 307.1, 307.51, 307.54, 327.2-327.8, 330-331.2, 331.5-337.9, 340-341.9, 345-345.91, 349-349.8, 353.6-353.9, 356-356.9, 357.0-357.7, 358-359.9, 376.0-376.1, 391-391.9, 392.0, 393-398.99, 402-404.93, 410-414.9, 416.1, 417-417.9, 420-423, 423.1-425.9, 427-427.32, 427.6-427.89, 429.0-429.1, 430-435.9, 437.0-437.2, 437.4-437.8, 441-443.9, 446-457.9, 459, 459.1-459.39, 470, 470.9-474.9, 476-476.1, 477-479, 490-504.9, 506-506.9, 508-509, 515, 516-517.8, 518.6-518.7, 518.9, 519.0-519.4, 530-536.1, 536.4-536.49, 537-537.6, 537.8-537.84, 538-543.9, 550-553.6, 555-558.9, 560-560.39, 560.8-560.9, 562-562.13, 564-564.7, 565-566.9, 569.0-569.44, 569.5-569.71, 569.84-569.85, 571-571.9, 572.3-572.9, 573.0-573.4, 573.8-577.9, 579-583.9, 585-585.9, 588-590.9, 592-593.89, 594-599.69, 599.8-599.89, 601-602.9, 604-604.99, 608.2-608.24, 610-610.9, 617-618.9, 620-620.9, 621.4-621.9, 622.3-622.7, 629-629.81, 680-689, 694-695.59, 707-707.9, 710-711.99, 714-714.33, 714.8-714.9, 728.86, 728.88, 730.1-730.19, 732-732.9, 733.0-733.19, 740-749.04, 749.2-758.9, 759.0-759.89, 760.7-760.79	C47-C54.9, C56-C57.8, C58-C58.0, C60-C63.8, C64-C67.9, C68.0-C68.8, C69-C75.8, C81-C86.6, C88-C96.9, D00.00-D00.2, D01.0-D01.3, D02.0-D02.3, D03-D06.9, D07.0-D07.2, D07.4-D07.5, D09.0, D09.2-D09.3, D09.8, D10.0-D10.7, D11-D12.9, D13.0-D13.7, D14.0-D14.32, D15-D16.9, D22-D27.9, D28.0-D28.7, D29.0-D29.8, D30.0-D30.8, D31-D36, D36.1-D36.7, D37.01-D37.5, D38.0-D38.5, D39.1-D39.2, D39.8, D40.0-D40.8, D41.0-D41.8, D42-D43.9, D44.0-D44.8, D45-D45.9, D47-D47.0, D47.2-D47.9, D48.0-D48.62, D49.2-D49.4, D49.6, D49.81, D52.1, D55-D58.9, D59.0-D59.3, D59.5-D59.6, D60-D61.9, D63.1, D64.0, D64.4, D66-D67, D68.0-D69.8, D70-D75.89, D76-D78.89, D86-D86.8, D86.82-D86.9, D89-D89.3, E03-E07.1, E09-E14.9, E15.0, E16.0-E16.9, E20-E34.8, E36-E36.8, E65-E68, E70-E85.29, E87.71, E88-E89.9, F00-F03.91, F06.2, F10-F16.99, F18-F29.9, F50.0-F50.5, G10-G13.8, G20-G26.0, G30-G31.9, G35-G37.9, G40-G41.9, G45-G46.8, G47.3-G47.39, G61-G61.9, G70-G73.7, G90-G90.9, G93.7, G95-G95.9, G97-G97.9, H05.0-H05.119, I01-I01.9, I02.0, I05-I09.9, I11-I13.9, I20-I25.9, I27.1, I28-I28.8, I30-I31.1, I31.8-I43.9, I47-I48.92, I51.0-I51.5, I60-I61.9, I62.0-I62.03, I63-I63.9, I65-I66.9, I67.0-I67.3, I67.5-I67.7, I68.0-I68.2, I69.0-I69.398, I70.2-I70.799, I71-I73.9, I77-I89.9, I91.9, I95.2-I95.3, I97-I98, I98.2, I98.9, J30-J35.9, J37-J47.9, J60-J63.8, J65-J68.9, J70-J70.9, J82, J84-J84.9, J91-J92.9, J95-J95.9, K20-K29.91, K31-K31.89, K35-K38.9, K40-K46.9, K50-K52.9, K55-K62.9, K63.5, K64-K64.9, K66.8, K67, K68-K68.9, K70-K70.9, K71.3-K71.51, K71.7, K72.1-K74.69, K74.9, K75.2-K77.8, K80-K83.9, K85-K86.9, K90-K91.9, K92.8-K92.89, K94-K95.89, L00-L05.92, L08-L08.9, L10-L14.0, L51-L51.9, L88-L89.95, L93-L93.2
Non-communicable diseases (continued)	1	775.0-775.3, 779.4-779.5, 780.57, 780.59, 780.62-780.63, 786.03, 787.1, 788.0, 790.2-790.22, 790.3, 798-798.0, E850-E850.29, E850.9-E854.39, E860-E860.19	L97-L98.499, M00-M03.0, M03.2-M03.6, M05-M09.8, M30-M36.8, M40-M43.19, M65-M65.08, M71.0-M71.19, M80-M82.8, M86.3-M86.49, M87-M87.19, M88-M89.09, M89.5-M89.59, M89.7-M89.9, N00-N08.8, N10-N12.9, N14-N16.8, N18-N18.9, N20-N23.0, N25-N32.0, N32.3-N32.4, N34-N34.3, N36-N36.9, N39-N39.2, N41-N41.9, N44-N44.04, N45-N45.9, N49-N49.9, N60-N60.99, N65-N65.1, N72-N72.0, N75-N77.8, N80-N81.9, N83-N83.9, N84.0-N84.1, N87-N87.9, N99-N99.9, P04.3-P04.49, P70.0-P70.2, P96.0-P96.2, P96.5, Q00-Q07.9, Q10.4-Q18.9, Q20-Q28.9, Q30-Q36, Q37-Q45.9, Q50-Q87.89, Q89-Q89.8, Q90-Q93.9, Q95-Q99.8, R50.2, R50.82-R50.83, R73-R73.9, R78.0-R78.5, R95, X45-X45.9

eTable 3: GBD cause list and associated ICD9 and ICD10 codes (continued).

Cause	Level	ICD9	ICD10
Neoplasms	2	140-148.9, 150-158.9, 160-164.9, 170-175.9, 180-183.8, 184.0-184.4, 184.8, 185-186.9, 187.1-187.8, 188-188.9, 189.0-189.8, 190-194.8, 200-208.92, 209.0-209.17, 209.21-209.27, 209.31-209.57, 209.61, 209.63-209.67, 210.0-210.9, 211.0-211.8, 212.0-212.8, 213-213.9, 217-217.8, 219.0, 220-220.9, 221.0-221.8, 222.0-222.8, 223.0-223.89, 224-228.9, 229.0, 229.8, 230.1-230.8, 231.0-231.2, 232-232.9, 233.0-233.2, 233.31-233.32, 233.4-233.5, 233.7, 234.0-234.8, 235.0, 235.4, 235.6-235.8, 236.1-236.2, 236.4-236.5, 236.7, 236.91-237.3, 237.5-237.9, 238.0-238.5, 239.2-239.4, 239.6, 569.0, 569.43-569.44, 569.84-569.85, 610-610.9	C0-C13.9, C15-C25.9, C3-C34.92, C37-C38.8, C4-C41.9, C43-C45.9, C47-C54.9, C56-C57.8, C58-C58.0, C60-C63.8, C64-C67.9, C68.0-C68.8, C69-C75.8, C81-C86.6, C88-C96.9, D00.00-D00.2, D01.0-D01.3, D02.0-D02.3, D03-D06.9, D07.0-D07.2, D07.4-D07.5, D09.0, D09.2-D09.3, D09.8, D10.0-D10.7, D11-D12.9, D13.0-D13.7, D14.0-D14.32, D15-D16.9, D22-D24.9, D26.0, D27-D27.9, D28.0-D28.1, D28.7, D29.0-D29.8, D30.0-D30.8, D31-D36, D36.1-D36.7, D37.01-D37.5, D38.0-D38.5, D39.1-D39.2, D39.8, D40.0-D40.8, D41.0-D41.8, D42-D43.9, D44.0-D44.8, D45-D45.9, D47-D47.0, D47.2-D47.9, D48.0-D48.62, D49.2-D49.4, D49.6, D49.81, K31.7, K62.0-K62.1, K63.5, N60-N60.99, N84.0-N84.1, N87-N87.9
Lip and oral cavity cancer	3-4	140-145.9, 210.0-210.6, 235.0	C0-C08.9, D00.00-D00.07, D10.0-D10.5, D11-D11.9, D37.01-D37.04, D37.09
Nasopharynx cancer	3-4	147-147.9, 210.7-210.9	C11-C11.9, D00.08, D10.6, D37.05
Other pharynx cancer	3-4	146-146.9, 148-148.9	C09-C10.9, C12-C13.9, D10.7
Esophageal cancer	3-4	150-150.9, 211.0, 230.1	C15-C15.9, D00.1, D13.0
Stomach cancer	3-4	151-151.9, 209.23, 209.63, 211.1, 230.2	C16-C16.9, D00.2, D13.1, D37.1
Colon and rectum cancer	3-4	153-154.9, 209.1-209.17, 209.5-209.57, 211.3-211.4, 230.3-230.6	C18-C21.9, D01.0-D01.3, D12-D12.9, D37.3-D37.5
Liver cancer	3-4	155-155.9, 211.5	C22-C22.9, D13.4
Gallbladder and biliary tract cancer	3-4	156-156.9, 209.25-209.27, 209.65-209.67	C23-C24.9, D13.5
Pancreatic cancer	3-4	157-157.9, 211.6-211.7	C25-C25.9, D13.6-D13.7
Larynx cancer	3-4	161-161.9, 212.1, 231.0, 235.6	C32-C32.9, D02.0, D14.1, D38.0
Tracheal, bronchus, and lung cancer	3-4	162-162.9, 209.21, 209.61, 212.2-212.3, 231.1-231.2, 235.7	C33-C34.92, D02.1-D02.3, D14.2-D14.32, D38.1
Malignant skin melanoma	3-4	172-172.9	C43-C43.9, D03-D03.9, D22-D23.9, D48.5
Non-melanoma skin cancer	3	173-173.99, 222.4, 232-232.9, 238.2	C44-C44.99, D04-D04.9, D49.2
Non-melanoma skin cancer (squamous-cell carcinoma)	4	173-173.99, 222.4, 232-232.9, 238.2	C44-C44.99, D04-D04.9, D49.2
Breast cancer	3-4	174-175.9, 217-217.8, 233.0, 238.3, 239.3, 610-610.9	C50-C50.929, D05-D05.92, D24-D24.9, D48.6-D48.62, D49.3, N60-N60.99
Cervical cancer	3-4	180-180.9, 219.0, 233.1	C53-C53.9, D06-D06.9, D26.0
Uterine cancer	3-4	182-182.8, 233.2	C54-C54.9, D07.0-D07.2, N87-N87.9
Ovarian cancer	3-4	183-183.0, 220-220.9, 236.2	C56-C56.9, D27-D27.9, D39.1-D39.12
Prostate cancer	3-4	185-185.9, 222.2, 236.5	C61-C61.9, D07.5, D29.1, D40.0
Testicular cancer	3-4	186-186.9, 222.0, 222.3, 236.4	C62-C62.92, D29.2-D29.8, D40.1-D40.8
Kidney cancer	3-4	189.0-189.1, 209.24, 209.64, 223.0-223.1, 236.91	C64-C65.9, D30.0-D30.12, D41.0-D41.12
Bladder cancer	3-4	188-188.9, 223.3, 233.7, 236.7, 239.4	C67-C67.9, D09.0, D30.3, D41.4-D41.8, D49.4
Brain and nervous system cancer	3-4	191-192.9	C70-C72.9
Thyroid cancer	3-4	193-193.9, 226-226.9	C73-C73.9, D09.3, D09.8, D34-D34.9, D44.0
Mesothelioma	3-4	158.9, 163-163.9, 212.4	C45-C45.9
Hodgkin lymphoma	3-4	201-201.98	C81-C81.99
Non-Hodgkin lymphoma	3-4	200-200.9, 202-202.98	C82-C86.6, C96-C96.9
Multiple myeloma	3-4	203-203.9	C88-C90.9
Leukemia	3	204-208.92	C91-C95.92
Acute lymphoid leukemia	4	204.0-204.02	C91.0-C91.02
Chronic lymphoid leukemia	4	204.1-204.12	C91.1-C91.12
Acute myeloid leukemia	4	205.0-205.02, 205.3-205.32, 206.0-206.02, 207.0	C92.0-C92.02, C92.3-C92.62, C93.0-C93.02, C94.0-C94.02, C94.2-C94.22, C94.4-C94.5
Chronic myeloid leukemia	4	205.1-205.12, 206.1-206.12, 207.1	C92.1-C92.12

eTable 3: GBD cause list and associated ICD9 and ICD10 codes (continued).

Cause	Level	ICD9	ICD10
Other neoplasms	3-4	152-152.9, 158-158.8, 160-160.9, 164-164.9, 170-171.9, 181-181.9, 182.9, 183.2-183.8, 184.0-184.4, 184.8, 187.1-187.8, 189.2-189.8, 190-190.9, 194-194.8, 209.0-209.03, 209.22, 209.31-209.43, 211.2, 211.8, 212.0, 212.5-212.8, 213-213.9, 221.0-221.8, 222.1, 222.8, 223.2, 223.8-223.89, 224-225.9, 227-228.9, 229.0, 229.8, 230.7-230.8, 233.31-233.32, 233.4-233.5, 234.0-234.8, 235.4, 235.8, 236.1, 236.99-237.3, 237.5-237.9, 238.0-238.1, 238.4-238.5, 239.2, 239.6, 569.0, 569.43-569.44, 569.84-569.85	C17-C17.9, C3-C31.9, C37-C38.8, C4-C41.9, C47-C5, C51-C52.9, C57-C57.8, C58-C58.0, C60-C60.9, C63-C63.8, C66-C66.9, C68.0-C68.8, C69-C7, C74-C75.8, D07.4, D09.2-D09.22, D13.2-D13.39, D14.0, D15-D16.9, D28.0-D28.1, D28.7, D29.0, D30.2-D30.22, D30.4-D30.8, D31-D33.9, D35-D36, D36.1-D36.7, D37.2, D38.2-D38.5, D39.2, D39.8, D41.2-D41.3, D42-D43.9, D44.1-D44.8, D45-D45.9, D47-D47.0, D47.2-D47.9, D48.0-D48.4, D49.6, D49.81, K31.7, K62.0-K62.1, K63.5, N84.0-N84.1
Cardiovascular diseases	2	036.41-036.43, 036.6, 074.2, 074.21-074.23, 391-391.9, 392.0, 393-398.99, 402-402.91, 410-414.9, 417-417.9, 420-423, 423.1-425.9, 427-427.32, 427.6-427.89, 429.0-429.1, 430-435.9, 437.0-437.2, 437.5-437.8, 441-443.9, 447-454.9, 456, 456.3-457.9, 459, 459.1-459.39	A39.5-A39.53, B33.2-B33.24, D86.85, G45-G46.8, I01-I01.9, I02.0, I05-I09.9, I11-I11.9, I20-I25.9, I28-I28.8, I30-I31.1, I31.8-I43.9, I47-I48.92, I51.0-I51.5, I60-I61.9, I62.0-I62.03, I63-I63.9, I65-I66.9, I67.0-I67.3, I67.5-I67.6, I68.0-I68.2, I69.0-I69.398, I70.2-I70.799, I71-I73.9, I77-I83.93, I86-I89.9, I91.9, I98
Rheumatic heart disease	3-4	391-391.9, 392.0, 393-398.99	I01-I01.9, I02.0, I05-I09.9
Ischemic heart disease	3-4	410-414.9	I20-I25.9
Cerebrovascular disease	3	430-435.9, 437.0-437.2, 437.5-437.8	G45-G46.8, I60-I61.9, I62.0-I62.03, I63-I63.9, I65-I66.9, I67.0-I67.3, I67.5-I67.6, I68.1-I68.2, I69.0-I69.398
Ischemic stroke	4	433-435.9, 437.0-437.1, 437.5-437.8	G45-G46.8, I63-I63.9, I65-I66.9, I67.2-I67.3, I67.5-I67.6, I69.3-I69.398
Hemorrhagic stroke	4	430-432.9, 437.2	I60-I61.9, I62.0-I62.03, I67.0-I67.1, I68.1-I68.2, I69.0-I69.298
Hypertensive heart disease	3-4	402-402.91	I11-I11.9
Cardiomyopathy and myocarditis	3-4	036.43, 036.6, 074.23, 422-422.99, 425-425.9, 429.0-429.1	A39.52, B33.2-B33.24, D86.85, I40-I43.9, I51.4-I51.5
Atrial fibrillation and flutter	3-4	427.3-427.32	I48-I48.92
Aortic aneurysm	3-4	441-441.9	I71-I71.9
Peripheral arterial disease	3-4	443.0-443.9	I70.2-I70.799, I73-I73.9
Endocarditis	3-4	036.42, 074.22, 421-421.9, 424.9-424.91	A39.51, I33-I33.9, I38-I39.9
Other cardiovascular and circulatory diseases	3-4	036.41, 074.2, 074.21, 417-417.9, 420-420.99, 423, 423.1-424.8, 424.99, 427-427.2, 427.6-427.89, 442-443, 447-454.9, 456, 456.3-457.9, 459, 459.1-459.39	A39.5-A39.50, A39.53, I28-I28.8, I30-I31.1, I31.8-I32.8, I34-I37.9, I47-I47.9, I51.0-I51.3, I68.0, I72-I72.9, I77-I83.93, I86-I89.9, I91.9, I98
Chronic respiratory diseases	2	135-135.9, 136.6, 327.2-327.8, 470, 470.9-474.9, 476-476.1, 477-479, 490-504.9, 506-506.9, 508-509, 515, 516-517.8, 518.6, 518.9, 519.1-519.4, 780.57, 786.03	D86-D86.2, D86.89-D86.9, G47.3-G47.39, J30-J35.9, J37-J47.9, J60-J63.8, J65-J68.9, J70-J70.1, J70.8-J70.9, J82, J84-J84.9, J91-J92.9
Chronic obstructive pulmonary disease	3-4	490-492.9, 494-494.9, 496-499	J40-J44.9, J47-J47.9
Pneumoconiosis	3	500-504.9	J60-J63.8, J65-J65.0, J92.0
Silicosis	4	502-502.9, 503.0, 503.9	J62-J62.9
Asbestosis	4	501	J61-J61.0, J92.0
Coal workers pneumoconiosis	4	500-500.9, 501.0-501.9	J60-J60.0
Other pneumoconiosis	4	503, 503.1, 504-504.9	J63-J63.8, J65-J65.0
Asthma	3-4	493-493.92	J45-J46.9
Interstitial lung disease and pulmonary sarcoidosis	3-4	135-135.9, 136.6, 515, 516-516.9	D86-D86.2, D86.89-D86.9, J84-J84.9
Other chronic respiratory diseases	3-4	327.2-327.8, 470, 470.9-474.9, 476-476.1, 477-479, 495-495.9, 506-506.9, 508-509, 517-517.8, 518.6, 518.9, 519.1-519.4, 780.57, 786.03	G47.3-G47.39, J30-J35.9, J37-J39.9, J66-J68.9, J70-J70.1, J70.8-J70.9, J82, J91-J92, J92.9
Cirrhosis and other chronic liver diseases	2-4	070.22-070.23, 070.32-070.33, 070.44, 070.54, 456.0-456.21, 571-571.9, 572.3-572.9, 573.0-573.3, 573.8-573.9	B18-B18.9, I85-I85.9, I98.2, K70-K70.9, K71.3-K71.51, K71.7, K72.1-K74.69, K74.9, K75.8-K76.0, K76.6-K76.7, K76.9

eTable 3: GBD cause list and associated ICD9 and ICD10 codes (continued).

Cause	Level	ICD9	ICD10
Digestive diseases	2	455-455.9, 530-536.1, 537-537.6, 537.8-537.84, 538, 540-543.9, 550-551.1, 551.3-552.1, 552.3-553.6, 555-558.9, 560-560.39, 560.8-560.9, 562-562.13, 564-564.1, 564.5-564.7, 565-566.9, 569.1-569.42, 569.5, 569.7-569.71, 573.4, 574-577.9, 579-579.2, 579.4-579.9, 787.1	I84-I84.9, K20-K29.91, K31-K31.6, K31.8-K31.89, K35-K38.9, K40-K42.9, K44-K46.9, K50-K52.9, K55-K62, K62.2-K62.6, K62.8-K62.9, K64-K64.9, K66.8, K67, K68-K68.9, K75.2-K75.4, K76.1-K76.5, K76.8-K76.89, K77-K77.8, K80-K83.9, K85-K86.9, K90-K90.9, K92.8-K92.89, M09.1
Peptic ulcer disease	3-4	531-534.91	K25-K28.9, K31, K31.1-K31.6, K31.8, K31.82-K31.89
Gastritis and duodenitis	3-4	535-535.9	K29-K29.91
Appendicitis	3-4	540-542.9	K35-K37.9, K38.3-K38.9
Paralytic ileus and intestinal obstruction	3-4	560-560.39, 560.8-560.9	K56-K56.9
Inguinal, femoral, and abdominal hernia	3-4	550-551.1, 551.3-552.1, 552.3-553.03, 553.6	K40-K42.9, K44-K46.9
Inflammatory bowel disease	3-4	555-556.9, 558-558.9, 569.5	K50-K52.9, M09.1
Vascular intestinal disorders	3-4	557-557.9	K55-K55.9
Gallbladder and biliary diseases	3-4	574-576.9	K80-K83.9
Pancreatitis	3-4	577-577.9, 579.4	K85-K86.9
Other digestive diseases	3-4	455-455.9, 530-530.9, 536-536.1, 537-537.6, 537.8-537.84, 538, 543-543.9, 553.1-553.3, 562-562.13, 564-564.1, 564.5-564.7, 565-566.9, 569.1-569.42, 569.7-569.71, 573.4, 579-579.2, 579.8-579.9, 787.1	I84-I84.9, K20-K24, K31.0, K31.81-K31.819, K38-K38.2, K57-K62, K62.2-K62.6, K62.8-K62.9, K64-K64.9, K66.8, K67, K68-K68.9, K75.2-K75.4, K76.1-K76.5, K76.8-K76.89, K77-K77.8, K90-K90.9, K92.8-K92.89
Neurological disorders	2	290-290.9, 294.1-294.9, 330-331.2, 331.5-337.9, 340-341.9, 345-345.91, 349, 349.2-349.8, 353.6-353.9, 356-356.9, 357.0-357.1, 357.3-357.4, 357.7, 358-359.9, 728.86, 728.88, 775.2	F00-F03.91, G10-G13.8, G20-G21.0, G21.2-G24, G24.1-G25.0, G25.2-G25.3, G25.5, G25.8-G26.0, G30-G31.1, G31.8-G31.9, G35-G37.9, G40-G41.9, G61-G61.9, G70-G72, G72.2-G73.7, G90-G90.9, G95-G95.9, M33-M33.99
Alzheimer disease and other dementias	3-4	290-290.9, 294.1-294.9, 331-331.2	F00-F03.91, G30-G31.1, G31.8-G31.9
Parkinson disease	3-4	332-332.9	G20-G21.0, G21.2-G22.0
Epilepsy	3-4	345-345.91	G40-G41.9
Multiple sclerosis	3-4	340-340.9	G35-G35.9
Motor neuron disease	3-4	335-335.29, 335.8-335.9	G12.2-G12.9
Other neurological disorders	3-4	330-330.9, 331.5-331.9, 333-334.9, 335.3, 336-337.9, 341-341.9, 349, 349.2-349.8, 353.6-353.9, 356-356.9, 357.0-357.1, 357.3-357.4, 357.7, 358-359.9, 728.86, 728.88, 775.2	G10-G12.1, G13-G13.8, G23-G24, G24.1-G25.0, G25.2-G25.3, G25.5, G25.8-G26.0, G36-G37.9, G61-G61.9, G70-G72, G72.2-G73.7, G90-G90.9, G95-G95.9, M33-M33.99
Mental and substance use disorders	2	291-292.9, 295-295.95, 303-303.93, 304.0-304.83, 305-305.93, 307.1, 307.51, 307.54, 357.5, 760.7-760.79, 780.59, 790.3, E850-E850.29, E850.9-E854.39, E860-E860.19	F06.2, F10-F16.99, F18-F29.9, F50.0-F50.5, G31.2, G72.1, P04.3-P04.49, P96.1, Q86.0, R78.0-R78.5, X45-X45.9
Schizophrenia	3-4	295-295.95	F06.2, F20-F23.9, F25-F29.9
Alcohol use disorders	3-4	291-291.9, 303-303.93, 305.0-305.03, 357.5, 790.3, E860-E860.19	F10-F10.99, G31.2, G72.1, P04.3, Q86.0, R78.0, X45-X45.9
Drug use disorders	3	292-292.9, 304.0-304.83, 305, 305.1-305.93, 760.7-760.79, E850-E850.29	F11-F16.99, F18-F19.99, P04.4-P04.49, P96.1, R78.1-R78.5
Opioid use disorders	4	304.0-304.03, 305.5-305.53, E850.0-E850.29	F11-F11.99, P96.1, R78.1
Cocaine use disorders	4	304.2-304.23, 305.6-305.63	F14-F14.99, R78.2
Amphetamine use disorders	4	304.4-304.43, 305.7-305.73	F15-F15.99
Other drug use disorders	4	292-292.9, 304.1-304.13, 304.5-304.83, 305, 305.1-305.13, 305.3-305.43, 305.8-305.93	F13-F13.99, F16-F16.99, F18-F19.99, R78.3-R78.5
Eating disorders	3	307.1, 307.51, 307.54	F50.0-F50.5
Anorexia nervosa	4	307.1, 307.54	F50.0-F50.1
Bulimia nervosa	4	307.51	F50.2-F50.5

eTable 3: GBD cause list and associated ICD9 and ICD10 codes (continued).

Cause	Level	ICD9	ICD10
Diabetes, urogenital, blood, and endocrine diseases	2	218-219, 219.1-219.9, 236.0, 240-243.9, 244.0-244.1, 244.3-244.8, 245-246.9, 250-259.9, 270-273.9, 275-276, 277-277.2, 277.4-277.9, 278.0-278.8, 282-284.9, 286-286.5, 286.7-289.7, 349.0-349.1, 357.2, 357.6, 403-404.93, 518.7, 519.0-519.09, 536.4-536.49, 539-539.9, 551.2-551.29, 552.2-552.29, 564.2-564.4, 569.6-569.69, 579.3, 580-583.9, 585-585.9, 588-590.9, 592-593.89, 594-599.69, 599.8-599.89, 601-602.9, 604-604.99, 608.2-608.24, 617-618.9, 620-620.9, 621.4-621.9, 622.3-622.7, 629-629.81, 775.0-775.1, 775.3, 779.4-779.5, 780.62-780.63, 788.0, 790.2-790.22	D25-D26, D26.1-D26.9, D28.2, D52.1, D55-D58.9, D59.0-D59.3, D59.5-D59.6, D60-D61.9, D63.1, D64.0, D64.4, D66-D67, D68.0-D69.8, D70-D75.89, D76-D78.89, D86.8, D86.82-D86.84, D86.86-D86.87, D89-D89.3, E03-E07.1, E09-E14.9, E15.0, E16.0-E16.9, E20-E34.8, E36-E36.8, E65-E68, E70-E85.29, E87.71, E88-E89.9, G21.1-G21.19, G24.0-G24.09, G25.1, G25.4, G25.6-G25.79, G72.0, G93.7, G97-G97.9, I12-I13.9, I95.2-I95.3, I97-I97.9, I98.9, J70.2-J70.5, J95-J95.9, K43-K43.9, K62.7, K91-K91.9, K94-K95.89, M87.1-M87.19, N00-N08.8, N10-N12.9, N14-N16.8, N18-N18.9, N20-N23.0, N25-N32.0, N32.3-N32.4, N34-N34.3, N36-N36.9, N39-N39.2, N41-N41.9, N44-N44.04, N45-N45.9, N49-N49.9, N65-N65.1, N72-N72.0, N75-N77.8, N80-N81.9, N83-N83.9, N99-N99.9, P70.0-P70.2, P96.2, P96.5, R50.2, R50.82-R50.83, R73-R73.9
Diabetes mellitus	3-4	250-250.39, 250.5-250.99, 357.2, 775.0-775.1, 790.2-790.22	E10-E10.11, E10.3-E11.1, E11.3-E12.1, E12.3-E13.11, E13.3-E14.1, E14.3-E14.9, P70.0-P70.2, R73-R73.9
Acute glomerulonephritis	3-4	580-580.9	N00-N01.9
Chronic kidney disease	3	250.4-250.49, 403-404.93, 581-583.9, 585-585.9, 589-589.9	D63.1, E10.2-E10.29, E11.2-E11.29, E12.2, E13.2-E13.29, E14.2, I12-I13.9, N02-N08.8, N15.0, N18-N18.9
Chronic kidney disease due to diabetes mellitus	4	250.4-250.49	E10.2-E10.29, E11.2-E11.29, E12.2, E13.2-E13.29, E14.2
Chronic kidney disease due to hypertension	4	403-404.93	I12-I13.9
Chronic kidney disease due to glomerulonephritis	4	581-583.9	N03-N06.9
Chronic kidney disease due to other causes	4	589-589.9	N02-N02.9, N07-N08.8, N15.0
Urinary diseases and male infertility	3	588-588.9, 590-590.9, 592-593.89, 594-596.81, 596.89-598.1, 598.8-599.69, 599.8-599.89, 601-602.9, 604-604.99, 608.2-608.24, 788.0	N10-N12.9, N15, N15.1-N16.8, N20-N23.0, N25-N32.0, N32.3-N32.4, N34-N34.3, N36-N36.9, N39-N39.2, N41-N41.9, N44-N44.04, N45-N45.9, N49-N49.9
Interstitial nephritis and urinary tract infections	4	590-590.9, 595-595.9, 597-597.9, 599.0	N10-N12.9, N15, N15.1-N16.8, N30-N30.91, N34-N34.3, N39.0-N39.2
Urolithiasis	4	592-592.9, 594-594.9, 788.0	N20-N23.0
Other urinary diseases	4	588-588.9, 593-593.89, 596-596.81, 596.89-596.9, 598-598.1, 598.8-599, 599.1-599.69, 599.8-599.89, 601-602.9, 604-604.99, 608.2-608.24	N25-N29.8, N31-N32.0, N32.3-N32.4, N36-N36.9, N39, N41-N41.9, N44-N44.04, N45-N45.9, N49-N49.9
Gynecological diseases	3	218-219, 219.1-219.9, 236.0, 256.4, 617-618.9, 620-620.9, 621.4-621.9, 622.3-622.7, 629-629.81	D25-D26, D26.1-D26.9, D28.2, E28.2, N72-N72.0, N75-N77.8, N80-N81.9, N83-N83.9
Uterine fibroids	4	218-219, 219.1-219.9, 236.0	D25-D26, D26.1-D26.9, D28.2
Polycystic ovarian syndrome	4	256.4	E28.2
Endometriosis	4	617-617.9	N80-N80.9
Genital prolapse	4	618-618.9	N81-N81.9
Other gynecological diseases	4	620-620.9, 621.4-621.9, 622.3-622.7, 629-629.81	N72-N72.0, N75-N77.8, N83-N83.9
Hemoglobinopathies and hemolytic anemias	3	282-284.9	D55-D58.9, D59.1, D59.3, D59.5, D60-D61.9, D64.0, D64.4
Thalassemias	4	282.4-282.49	D56-D56.9
Sickle cell disorders	4	282.6-282.68	D57-D57.219, D57.4-D57.819
G6PD deficiency	4	282.2-282.3	D55-D55.2
Other hemoglobinopathies and hemolytic anemias	4	282-282.1, 282.69-284.9	D55.3-D55.9, D58-D58.9, D59.1, D59.3, D59.5, D60-D61.9, D64.0, D64.4

eTable 3: GBD cause list and associated ICD9 and ICD10 codes (continued).

Cause	Level	ICD9	ICD10
Endocrine, metabolic, blood, and immune disorders	3-4	240-243.9, 244.0-244.1, 244.3-244.8, 245-246.9, 251-256.39, 256.8-259.9, 270-273.9, 275-276, 277-277.2, 277.4-277.9, 278.0-278.8, 286-286.5, 286.7-289.7, 349.0-349.1, 357.6, 518.7, 519.0-519.09, 536.4-536.49, 539-539.9, 551.2-551.29, 552.2-552.29, 564.2-564.4, 569.6-569.69, 579.3, 596.82-596.83, 598.2, 775.3, 779.4-779.5, 780.62-780.63	D52.1, D59.0, D59.2, D59.6, D66-D67, D68.0-D69.8, D70-D75.89, D76-D78.89, D86.8, D86.82-D86.84, D86.86-D86.87, D89-D89.3, E03-E07.1, E09-E09.9, E15.0, E16.0-E16.9, E20-E28.1, E28.3-E34.8, E36-E36.8, E65-E68, E70-E85.29, E87.71, E88-E89.9, G21.1-G21.19, G24.0-G24.09, G25.1, G25.4, G25.6-G25.79, G72.0, G93.7, G97-G97.9, I95.2-I95.3, I97-I97.9, I98.9, J70.2-J70.5, J95-J95.9, K43-K43.9, K62.7, K91-K91.9, K94-K95.89, M87.1-M87.19, N14-N14.4, N65-N65.1, N99-N99.9, P96.2, P96.5, R50.2, R50.82-R50.83
Musculoskeletal disorders	2	416.1, 437.4, 446-446.9, 695.4-695.59, 710-711.99, 714-714.33, 714.8-714.9, 730.1-730.19, 732-732.9, 733.0-733.19	I27.1, I67.7, L93-L93.2, M00-M03.0, M03.2-M03.6, M05-M09.0, M09.2-M09.8, M30-M32.9, M34-M36.8, M40-M43.19, M65-M65.08, M71.0-M71.19, M80-M82.8, M86.3-M86.49, M87-M87.09, M88-M89.09, M89.5-M89.59, M89.7-M89.9
Rheumatoid arthritis	3-4	714-714.33, 714.8-714.9	M05-M06.9, M08.0-M08.89
Other musculoskeletal disorders	3-4	416.1, 437.4, 446-446.9, 695.4-695.59, 710-711.99, 730.1-730.19, 732-732.9, 733.0-733.19	I27.1, I67.7, L93-L93.2, M00-M03.0, M03.2-M03.6, M07-M08, M08.9-M09.0, M09.2-M09.8, M30-M32.9, M34-M36.8, M40-M43.19, M65-M65.08, M71.0-M71.19, M80-M82.8, M86.3-M86.49, M87-M87.09, M88-M89.09, M89.5-M89.59, M89.7-M89.9
Other non-communicable diseases	2	035-035.9, 102-103.9, 133-133.6, 376.0-376.1, 680-689, 694-695.3, 707-707.9, 740-749.04, 749.2-758.9, 759.0-759.89, 798-798.0	A46-A46.0, A66-A67.9, B86, D86.3, H05.0-H05.119, L00-L05.92, L08-L08.9, L10-L14.0, L51-L51.9, L88-L89.95, L97-L98.499, P96.0, Q00-Q07.9, Q10.4-Q18.9, Q20-Q28.9, Q30-Q36, Q37-Q45.9, Q50-Q86, Q86.1-Q87.89, Q89-Q89.8, Q90-Q93.9, Q95-Q99.8, R95
Congenital anomalies	3	740-749.04, 749.2-758.9, 759.0-759.89	P96.0, Q00-Q07.9, Q10.4-Q18.9, Q20-Q28.9, Q30-Q36, Q37-Q45.9, Q50-Q86, Q86.1-Q87.89, Q89-Q89.8, Q90-Q93.9, Q95-Q99.8
Neural tube defects	4	740-741.93, 742.0	Q00-Q01.9, Q05-Q05.9
Congenital heart anomalies	4	745-747.9	Q20-Q28.9
Cleft lip and cleft palate	4	749-749.04, 749.2-749.25	Q35-Q36, Q37-Q37.9
Down syndrome	4	758	Q90-Q90.9
Other chromosomal abnormalities	4	758, 758.1-758.6, 758.8-758.9	Q91-Q93.9, Q95-Q95.9, Q97-Q97.9, Q99-Q99.8
Other congenital anomalies	4	742, 742.1-744.9, 748-748.9, 749.6-757.9, 759.0-759.89	P96.0, Q02-Q04.9, Q06-Q07.9, Q10.4-Q18.9, Q30-Q34.9, Q38-Q45.9, Q50-Q86, Q86.1-Q87.89, Q89-Q89.8
Skin and subcutaneous diseases	3	035-035.9, 102-103.9, 133-133.6, 680-689, 694-695.3, 707-707.9	A46-A46.0, A66-A67.9, B86, D86.3, L00-L05.92, L08-L08.9, L10-L14.0, L51-L51.9, L88-L89.95, L97-L98.499
Cellulitis	4	681-682.9	L03-L03.91
Pyoderma	4	035-035.9, 102-103.9, 680-680.9, 683-689	A46-A46.0, A66-A67.9, L00-L02.93, L04-L05.92, L08-L08.9, L88, L97-L98.499
Decubitus ulcer	4	707-707.9	L89-L89.95
Other skin and subcutaneous diseases	4	694-695.3	D86.3, L10-L14.0, L51-L51.9
Sudden infant death syndrome	3-4	798-798.0	R95

eTable 3: GBD cause list and associated ICD9 and ICD10 codes (continued).

Cause	Level	ICD9	ICD10
Injuries	1	E800-E800.3, E801-E801.3, E802-E802.3, E803-E803.3, E804-E804.3, E805-E805.3, E806-E806.3, E807-E807.3, E810.0-E810.7, E811.0-E811.7, E812.0-E812.7, E813.0-E813.7, E814.0-E814.7, E815.0-E815.7, E816.0-E816.7, E817.0-E817.7, E818.0-E818.7, E819.0-E819.7, E820.0-E820.7, E821.0-E821.7, E822.0-E822.7, E823.0-E823.7, E824.0-E824.7, E825.0-E825.7, E826.0-E826.4, E827.0-E827.4, E828.0-E828.4, E829.0-E829.4, E830-E838.9, E840-E849.9, E850.3-E850.89, E854.8, E856-E857.09, E860.2-E869.99, E870-E876.9, E878-E879.9, E880-E886.99, E888-E928.89, E929.1-E929.5, E930-E979.9, E990-E999.1	V00-V86.99, V87.2-V87.3, V88.2-V88.3, V90-V98.8, W00-W46.2, W49-W62.9, W64-W70.9, W73-W75.9, W77-W81.9, W83-W94.9, W97.9, W99-X06.9, X08-X39.9, X46-X47, X47.1-X47.8, X48-X48.9, X50-X54.9, X57-X58.9, X60-Y08.9, Y35-Y84.9, Y87.0-Y87.1, Y88-Y88.3, Y89.0-Y89.1
Transport injuries	2	E800-E800.3, E801-E801.3, E802-E802.3, E803-E803.3, E804-E804.3, E805-E805.3, E806-E806.3, E807-E807.3, E810.0-E810.7, E811.0-E811.7, E812.0-E812.7, E813.0-E813.7, E814.0-E814.7, E815.0-E815.7, E816.0-E816.7, E817.0-E817.7, E818.0-E818.7, E819.0-E819.7, E820.0-E820.7, E821.0-E821.7, E822.0-E822.7, E823.0-E823.7, E824.0-E824.7, E825.0-E825.7, E826.0-E826.4, E827.0-E827.4, E828.0-E828.4, E829.0-E829.4, E830-E838.9, E840-E849.9, E929.1	V00-V86.99, V87.2-V87.3, V88.2-V88.3, V90-V98.8
Road injuries	3	E800.3, E801.3, E802.3, E803.3, E804.3, E805.3, E806.3, E807.3, E810.0-E810.6, E811.0-E811.7, E812.0-E812.7, E813.0-E813.7, E814.0-E814.7, E815.0-E815.7, E816.0-E816.7, E817.0-E817.7, E818.0-E818.7, E819.0-E819.7, E820.0-E820.6, E821.0-E821.6, E822.0-E822.7, E823.0-E823.7, E824.0-E824.7, E825.0-E825.7, E826.0-E826.1, E826.3-E826.4, E827.0, E827.3-E827.4, E828.0, E828.4, E829.0-E829.4	V01-V04.99, V06-V80.929, V82-V82.9, V87.2-V87.3
Pedestrian road injuries	4	E811.7, E812.7, E813.7, E814.7, E815.7, E816.7, E817.7, E818.7, E819.7, E822.7, E823.7, E824.7, E825.7, E826.0, E827.0, E828.0, E829.0	V01-V04.99, V06-V09.9
Cyclist road injuries	4	E800.3, E801.3, E802.3, E803.3, E804.3, E805.3, E806.3, E807.3, E810.6, E811.6, E812.6, E813.6, E814.6, E815.6, E816.6, E817.6, E818.6, E819.6, E820.6, E821.6, E822.6, E823.6, E824.6, E825.6, E826.1	V10-V19.9
Motorcyclist road injuries	4	E810.2-E810.3, E811.2-E811.3, E812.2-E812.3, E813.2-E813.3, E814.2-E814.3, E815.2-E815.3, E816.2-E816.3, E817.2-E817.3, E818.2-E818.3, E819.2-E819.3, E820.2-E820.3, E821.2-E821.3, E822.2-E822.3, E823.2-E823.3, E824.2-E824.3, E825.2-E825.3	V20-V29.9
Motor vehicle road injuries	4	E810.0-E810.1, E811.0-E811.1, E812.0-E812.1, E813.0-E813.1, E814.0-E814.1, E815.0-E815.1, E816.0-E816.1, E817.0-E817.1, E818.0-E818.1, E819.0-E819.1, E820.0-E820.1, E821.0-E821.1, E822.0-E822.1, E823.0-E823.1, E824.0-E824.1, E825.0-E825.1	V30-V79.9, V87.2-V87.3

eTable 3: GBD cause list and associated ICD9 and ICD10 codes (continued).

Cause	Level	ICD9	ICD10
Other road injuries	4	E810.4-E810.5, E811.4-E811.5, E812.4-E812.5, E813.4-E813.5, E814.4-E814.5, E815.4-E815.5, E816.4-E816.5, E817.4-E817.5, E818.4-E818.5, E819.4-E819.5, E820.4-E820.5, E821.4-E821.5, E822.4-E822.5, E823.4-E823.5, E824.4-E824.5, E825.4-E825.5, E826.3-E826.4, E827.3-E827.4, E828.4, E829.4	V80-V80.929, V82-V82.9
Other transport injuries	3-4	E800-E800.2, E801-E801.2, E802-E802.2, E803-E803.2, E804-E804.2, E805-E805.2, E806-E806.2, E807-E807.2, E810.7, E820.7, E821.7, E826.2, E827.2, E828.2, E830-E838.9, E840-E849.9, E929.1	V00-V00.898, V05-V05.99, V81-V81.9, V83-V86.99, V88.2-V88.3, V90-V98.8
Unintentional injuries	2	E850.3-E850.89, E854.8, E856-E857.09, E860.2-E869.99, E870-E876.9, E878-E879.9, E880-E886.99, E888-E906.99, E910-E928.89, E929.2-E929.5, E930-E949.9	W00-W46.2, W49-W62.9, W64-W70.9, W73-W75.9, W77-W81.9, W83-W94.9, W97.9, W99-X06.9, X08-X32.9, X39-X39.9, X46-X47, X47.1-X47.8, X48-X48.9, X50-X54.9, X57-X58.9, Y38.9-Y84.9, Y88-Y88.3
Falls	3-4	E880-E886.99, E888-E888.9, E929.3	W00-W19.9
Drowning	3-4	E910-E910.99	W65-W70.9, W73-W74.9
Fire, heat, and hot substances	3-4	E890-E899.09, E924-E924.99, E929.4	X00-X06.9, X08-X19.9
Poisonings	3-4	E850.3-E850.89, E854.8, E856-E857.09, E860.2-E869.99, E929.2	X46-X47, X47.1-X47.8, X48-X48.9
Exposure to mechanical forces	3	E913-E913.19, E916-E922.99, E928.1-E928.7	W20-W38.9, W40-W43.9, W45.0-W45.2, W46-W46.2, W49-W52, W75-W75.9
Unintentional firearm injuries	4	E922-E922.99, E928.7	W32-W34.9
Unintentional suffocation	4	E913-E913.19	W75-W75.9
Other exposure to mechanical forces	4	E916-E921.99, E928.1-E928.6	W20-W31.9, W35-W38.9, W40-W43.9, W45.0-W45.2, W46-W46.2, W49-W52
Adverse effects of medical treatment	3-4	E870-E876.9, E878-E879.9, E930-E949.9	Y38.9-Y84.9, Y88-Y88.3
Animal contact	3	E905-E906.99	W52.0-W62.9, W64-W64.9, X20-X29.9
Venomous animal contact	4	E905-E905.99	X20-X29.9
Non-venomous animal contact	4	E906-E906.99	W52.0-W62.9, W64-W64.9
Foreign body	3	E911-E912.09, E913.8-E915.09	W44-W45, W45.3-W45.9, W78-W80.9, W83-W84.9
Pulmonary aspiration and foreign body in airway	4	E911-E912.09, E913.8-E913.99	W78-W80.9, W83-W84.9
Foreign body in other body part	4	E914-E915.09	W44-W45, W45.3-W45.9
Environmental heat and cold exposure	3-4	E900-E902.99, E926-E926.99, E929.5	W88-W94.9, W97.9, W99-W99.9, X30-X32.9, X39-X39.9
Other unintentional injuries	3-4	E903-E904.99, E913.2-E913.39, E923-E923.99, E925-E925.99, E927-E928.09, E928.8-E928.89	W39-W39.9, W77-W77.9, W81-W81.9, W85-W87.9, X50-X54.9, X57-X58.9
Self-harm and interpersonal violence	2	E950-E969	X60-Y08.9, Y87.0-Y87.1
Self-harm	3-4	E950-E959	X60-X84.9, Y87.0
Interpersonal violence	3	E960-E969	X85-Y08.9, Y87.1
Assault by firearm	4	E965-E965.4	X93-X94.0, X94.3-X94.7, X94.9-X95.9, X96.5
Assault by sharp object	4	E966	X99-X99.9
Assault by other means	4	E960-E964, E965.5-E965.9, E967-E969	X85-X92.9, X94.1-X94.2, X94.8, X96-X96.4, X96.6-X98.9, Y00-Y08.9, Y87.1
Forces of nature, war, and legal intervention	2	E907-E909.9, E970-E979.9, E990-E999.1	X33-X38.9, Y35-Y38.893, Y89.0-Y89.1
Exposure to forces of nature	3-4	E907-E909.9	X33-X38.9
Collective violence and legal intervention	3-4	E970-E979.9, E990-E999.1	Y35-Y38.893, Y89.0-Y89.1

eTable 3: GBD cause list and associated ICD9 and ICD10 codes (continued).

Cause	Level	ICD9	ICD10
Garbage Code		000-000.9, 002, 031-031.9, 038-038.9, 039.6, 040.0, 041.1-041.9, 067-069, 076-078.3, 078.8-078.9, 079.8-079.99, 084, 084.6, 085, 085.1-085.9, 089-089.9, 105-119, 125-125.3, 126-126.9, 127.2-127.9, 130-132.9, 133.8-134.9, 136.3-136.5, 136.8-136.9, 139.1-139.9, 149-149.9, 159-159.9, 165-169, 176-179.9, 183.9-184, 184.5, 184.9, 187, 187.9, 189, 189.9, 194.9-199.9, 209, 209.2-209.20, 209.29-209.30, 209.6-209.60, 209.62, 209.69-210, 211, 211.9-212, 212.9, 214-216.9, 221, 221.9-222, 222.9-223, 223.9, 229, 229.1, 229.9-230.0, 230.9-231, 231.8-231.9, 233, 233.3-233.30, 233.39, 233.6, 233.9-234, 234.9-235, 235.1-235.3, 235.5, 235.9-236, 236.3, 236.6, 236.9-236.90, 237.4, 238, 238.6-239.1, 239.5, 239.7-239.9, 244, 244.9, 247-249.91, 264-264.9, 274-274.9, 276.0-276.9, 277.3-277.39, 278, 279-280.0, 280.9-281, 285-285.9, 286.6, 289.8-289.9, 293-294.0, 296-302.9, 304, 304.9-304.93, 306-307.0, 307.2-307.50, 307.52-307.53, 307.59-320, 320.9, 324-327.19, 328-329, 331.3-331.4, 338-339.89, 342-344.9, 346-348.9, 349.81-353.5, 354-355.9, 357, 357.8-357.9, 360-376, 376.10-380.9, 384-389.9, 399-401.9, 405-409.4, 415-416.0, 416.2-416.9, 418-419.9, 423.0, 426-426.9, 427.4-427.5, 427.9-429, 429.2-429.9, 436-437, 437.3, 437.9-440.9, 444-445.89, 458-458.9, 459.0, 459.5-460.9, 462-464, 464.00, 464.1-464.10, 464.20, 464.3-464.30, 464.5-464.51, 465-465.9, 482.9-483, 484, 484.8-486.9, 505-505.9, 507-507.9, 510-514.9, 515.0-515.9, 518-518.53, 518.8-518.89, 519, 519.8-529.9, 536.2-536.3, 536.8-536.9, 537.7, 537.89-537.9, 544-549, 553.8-553.9, 559-559.0, 560.4-560.7, 561, 562.2-563, 564.8-564.9, 567-569, 569.49, 569.79-569.83, 569.86-570.9, 572-572.2, 573	A01, A14.9, A29, A31-A31.9, A40-A45.9, A47-A48.0, A48.3, A48.8-A49.02, A49.2-A49.9, A59-A59.9, A61-A62, A64-A64.0, A71-A73, A74.0, A76, A97, A99-A99.0, B07-B09, B11-B14, B28-B29, B30-B32.4, B34-B46.9, B49-B49.9, B54-B55, B55.1-B55.9, B58-B59.9, B61-B62, B64, B68-B68.9, B73-B74.2, B76-B76.9, B78-B82.9, B83.9-B85.4, B87-B89, B93-B94.0, B94.8-B94.9, B95.6-B99.9, C14-C14.9, C26-C29, C35-C36, C39-C39.9, C42, C46-C46.9, C55-C55.9, C57.9, C59-C6, C63.9, C68, C68.9, C75.9-C80.9, C87, C97-D00.0, D01, D01.4-D02, D02.4-D02.9, D07, D07.3-D07.39, D07.6-D09, D09.1-D09.19, D09.7, D09.9-D10, D10.9, D13, D13.9-D14, D14.4, D17-D21.9, D28, D28.9-D29, D29.9-D30, D30.9, D36.0, D36.9-D37.0, D37.6-D38, D38.6-D39.0, D39.7, D39.9-D40, D40.9-D41, D41.9, D44, D44.9, D46-D46.9, D47.1, D48, D48.7-D49.1, D49.5, D49.7-D49.8, D49.89-D50.0, D50.9, D54, D59, D59.4, D59.8-D59.9, D62-D63.0, D63.8-D64, D64.1-D64.2, D64.8-D65.9, D68, D69.9, D75.9, D79-D85, D87-D88, D89.8-D99, E07.8-E08.9, E15, E16, E17-E19, E34.9-E35.8, E37-E39, E47-E50.9, E62, E64.1, E69, E85.3-E87.70, E87.79-E87.99, E90-E998, F04-F06.1, F06.3-F07.0, F07.2-F09.9, F17-F17.9, F30-F50, F50.8-G00, G00.9-G02.8, G03.9, G06-G09.9, G15-G19, G27-G29, G32-G34, G38-G39, G42-G44.89, G47-G47.29, G47.4-G60.9, G62-G69, G74-G89.4, G91-G93.6, G93.8-G94.8, G96-G96.9, G98-H05, H05.12-H69.93, H71-H99, I00.0, I03-I04, I10-I10.9, I14-I19, I26-I27.0, I27.2-I27.9, I28.9-I29.9, I31.2-I31.4, I44-I46.9, I49-I51, I51.6-I59, I62, I62.1-I62.9, I64-I64.9, I67, I67.4, I67.8-I68

eTable 3: GBD cause list and associated ICD9 and ICD10 codes (continued).

Cause	Level	ICD9	ICD10
Garbage Code (continued)		573.5, 578-578.9, 584-584.9, 586-587.9, 591-591.9, 593.9, 599.7-599.72, 599.9-600.91, 603-603.9, 605-608.1, 608.3-609, 611-612.1, 615-616.9, 619-619.9, 621-621.35, 622-622.2, 622.8-628.9, 629.89-629.9, 637-637.92, 639-639.9, 690-693.9, 695.8-706.9, 708-709.9, 712-713.8, 714.4, 715-716, 716.1-728.85, 728.87, 728.89-730.09, 730.2-730.39, 730.7-731.9, 733, 733.2-739.9, 749.1-749.14, 759, 759.9, 770.0, 779.9-780.56, 780.58, 780.6-780.61, 780.64-786.02, 786.04-787.04, 787.2-787.9, 787.99-788, 788.1-790.1, 790.29, 790.4-797.9, 798.1-E80, E800.8-E800.9, E801.8-E801.9, E802.8-E802.9, E803.8-E803.9, E804.8-E804.9, E805.8-E805.9, E806.8-E806.9, E807.8-E810, E810.8-E811, E811.8-E812, E812.8-E813, E813.8-E814, E814.8-E815, E815.8-E816, E816.8-E817, E817.8-E818, E818.8-E819, E819.8-E820, E820.8-E821, E821.8-E822, E822.8-E823, E823.8-E824, E824.8-E825, E825.8-E826, E826.8-E827, E827.8-E828, E828.8-E829, E829.8-E83, E839, E85, E855-E855.99, E858-E859, E87, E877, E88, E887-E887.09, E928.9-E929.0, E929.8-E929.9, E980-E989	I68.8-I69, I69.4-I70.1, I70.8-I70.92, I74-I76, I90, I92-I95.1, I95.8-I96.9, I98.4-I98.8, I99-J00.0, J02, J02.8-J03, J03.8-J04, J04.1-J04.31, J05.1-J05.10, J06-J08, J15.9, J17-J19.6, J22-J29, J48-J59, J64-J64.9, J69-J69.9, J71-J81.9, J83, J85-J90.9, J93-J94.9, J96-K19, K30, K31.9-K34, K39, K47-K49, K53-K54, K63-K63.4, K63.8-K63.9, K65-K66.1, K66.9, K69, K71-K71.2, K71.6, K71.8-K72.01, K75-K75.1, K78-K79, K84, K87-K89, K92-K92.2, K92.9-K93, K93.1-K93.8, K96-K99, L06-L07, L09, L15-L50.9, L52-L87.9, L90-L92.9, L94-L96, L98.5-L99.8, M04, M10-M12.09, M12.2-M29, M37-M39, M43.2-M49, M49.2-M64, M65.1-M71, M71.2-M73, M73.8-M79.9, M83-M86.29, M86.5-M86.9, M87.2-M87.9, M89.1-M89.49, M90-M99.9, N09, N13-N13.9, N17-N17.9, N19-N19.9, N24, N32.1-N32.2, N32.8-N33.8, N35-N35.9, N37-N38, N39.3-N40.9, N42-N43.42, N44.1-N44.8, N46-N48.9, N50-N59, N61-N64.9, N66-N69, N78-N79, N82-N82.9, N84, N84.2-N86, N88-N95.9, N97-N97.9, O08-O08.9, O17-O19, O27, O37-O39, O49-O59, O78-O79, O93-O95.9, P06, P16-P18, P23, P23.5-P23.9, P30-P34.2, P37.3-P37.4, P40-P49, P62-P69, P73, P79, P82, P85-P89, P96.9-P99.9, Q08-Q10.3, Q19, Q29, Q36.0-Q36.9, Q46-Q49, Q88, Q89.9, Q94, Q99.9-R19.6, R19.8-R50.1, R50.8-R50.81, R50.84-R72.9, R74-R78, R78.6-R94.8, R95.0-T71.161, T71.163-U03, U05-U99, V87-V87.1, V87.4-V88.1, V88.4-V89.9, V99-V99.0, W47-W48, W63, W71-W72, W76-W76.9, W82, W95-W97, W98, X07, X40-X44.9, X47.0, X47.9, X49-X49.9, X55-X56, X59-X59.9, Y09-Y34.9, Y85-Y87, Y87.2, Y89, Y89.9-Y99.9

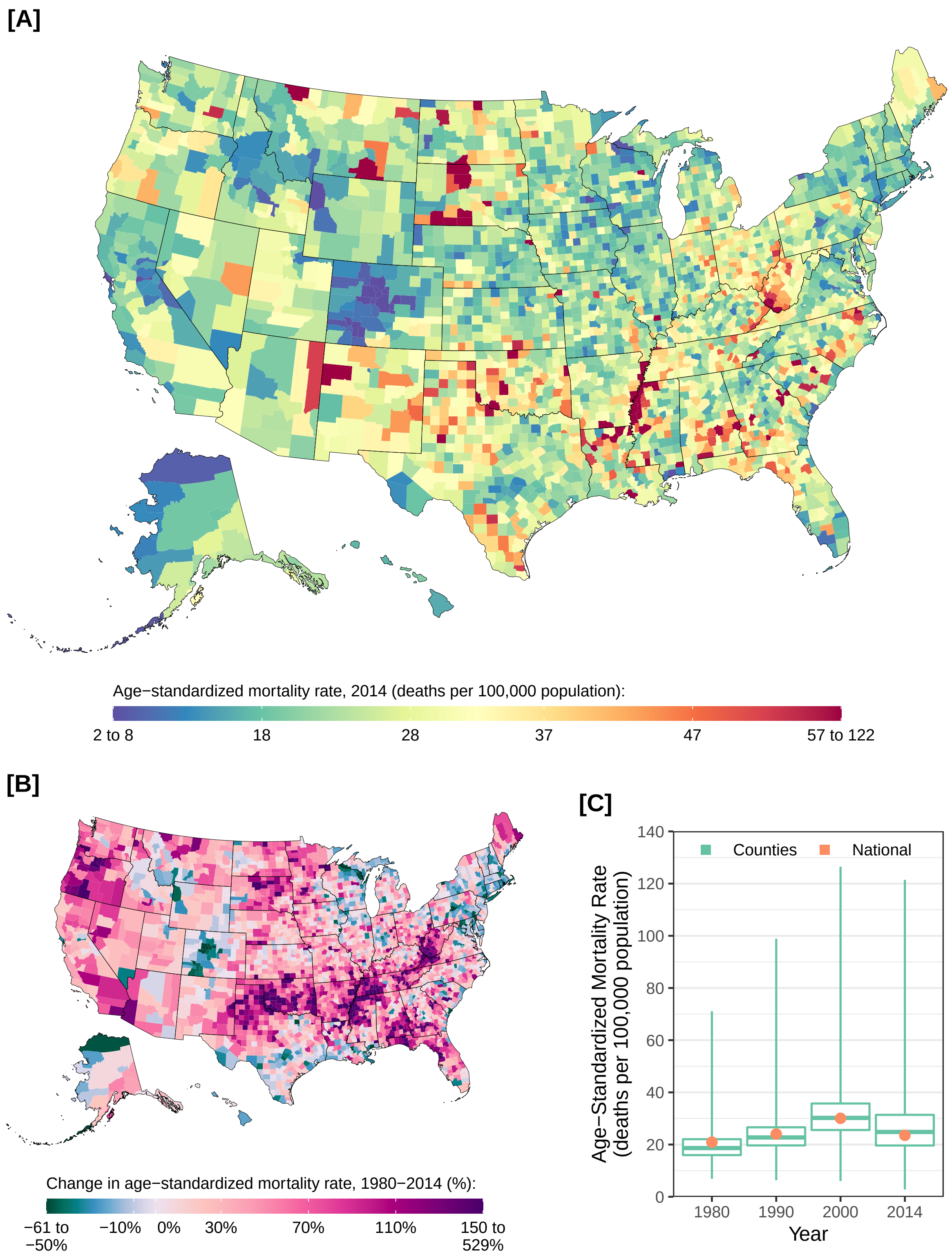

eFigure 1: County-level mortality among males from diabetes mellitus. [A] Age-standardized mortality rate in 2014; [B] Relative change in the age-standardized mortality rate between 1980 and 2014; [C] Age-standardized mortality rate in 1980, 1990, 2000, and 2014. In panel [C], the boxes indicate the 25th, 50th, and 75th percentile across all counties while the lines indicate the full range across counties and the dots indicate the national-level rate.

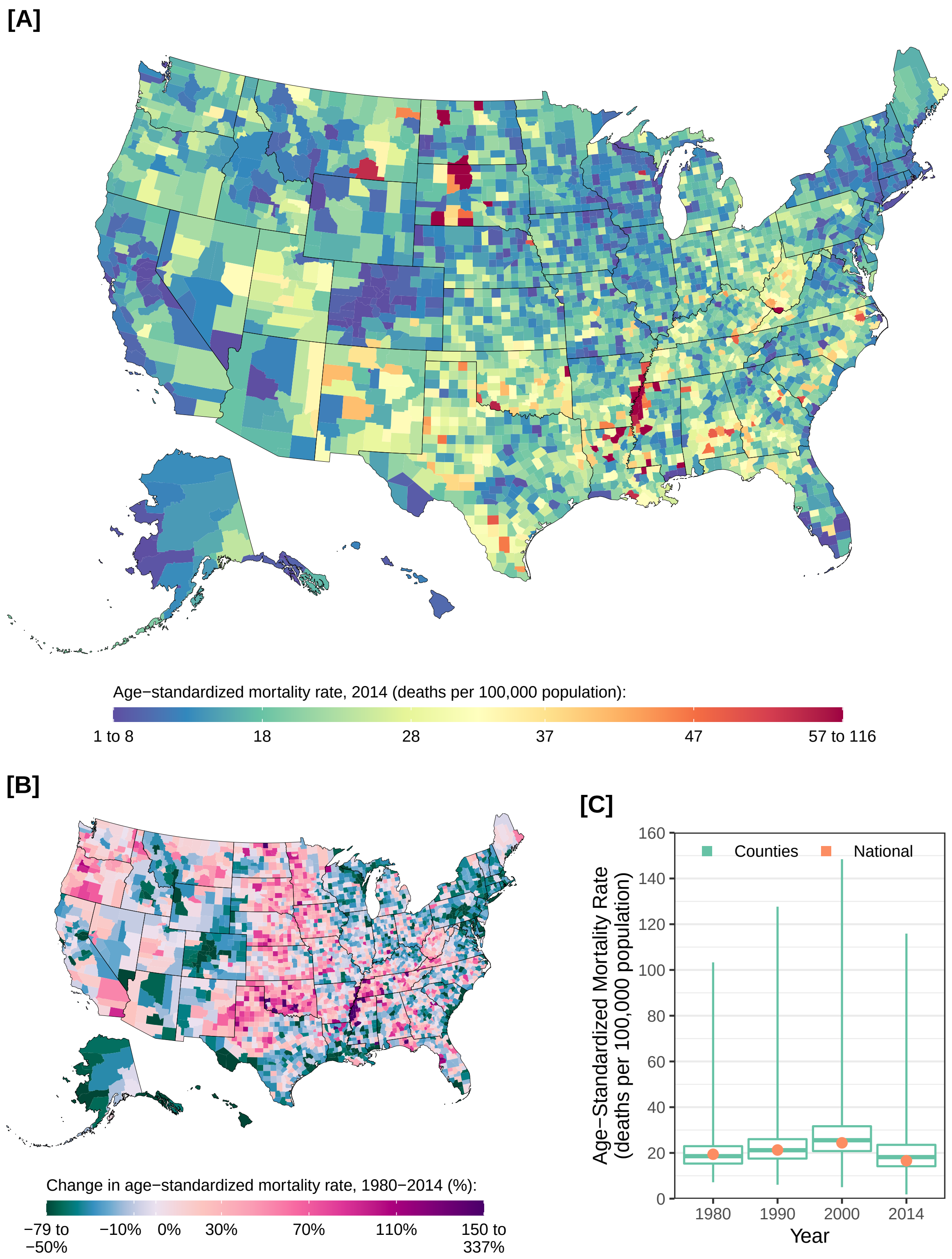

eFigure 2: County-level mortality among females from diabetes mellitus. [A] Age-standardized mortality rate in 2014; [B] Relative change in the age-standardized mortality rate between 1980 and 2014; [C] Age-standardized mortality rate in 1980, 1990, 2000, and 2014. In panel [C], the boxes indicate the 25th, 50th, and 75th percentile across all counties while the lines indicate the full range across counties and the dots indicate the national-level rate.

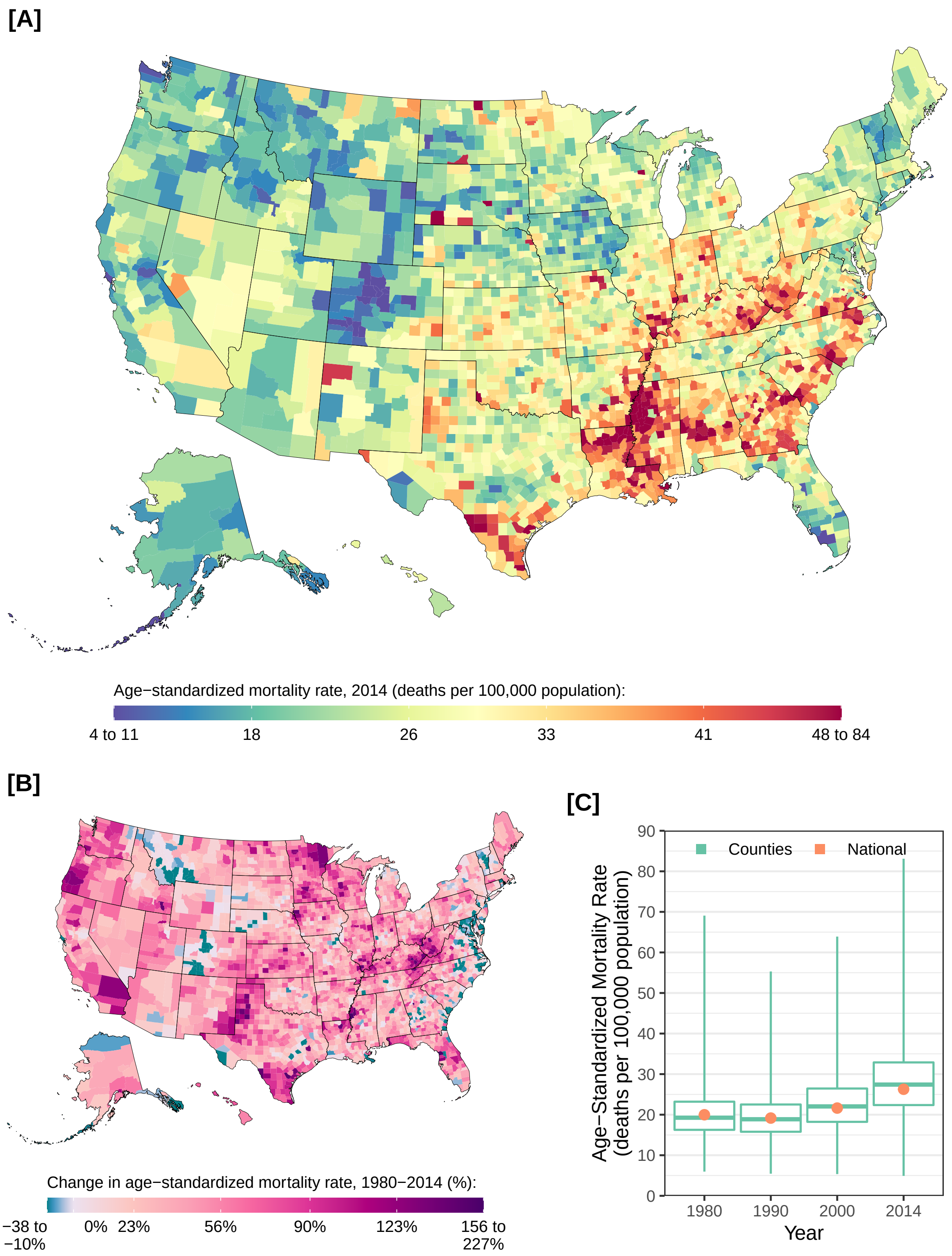

eFigure 3: County-level mortality among males from chronic kidney disease. [A] Age-standardized mortality rate in 2014; [B] Relative change in the age-standardized mortality rate between 1980 and 2014; [C] Age-standardized mortality rate in 1980, 1990, 2000, and 2014. In panel [C], the boxes indicate the 25th, 50th, and 75th percentile across all counties while the lines indicate the full range across counties and the dots indicate the national-level rate.

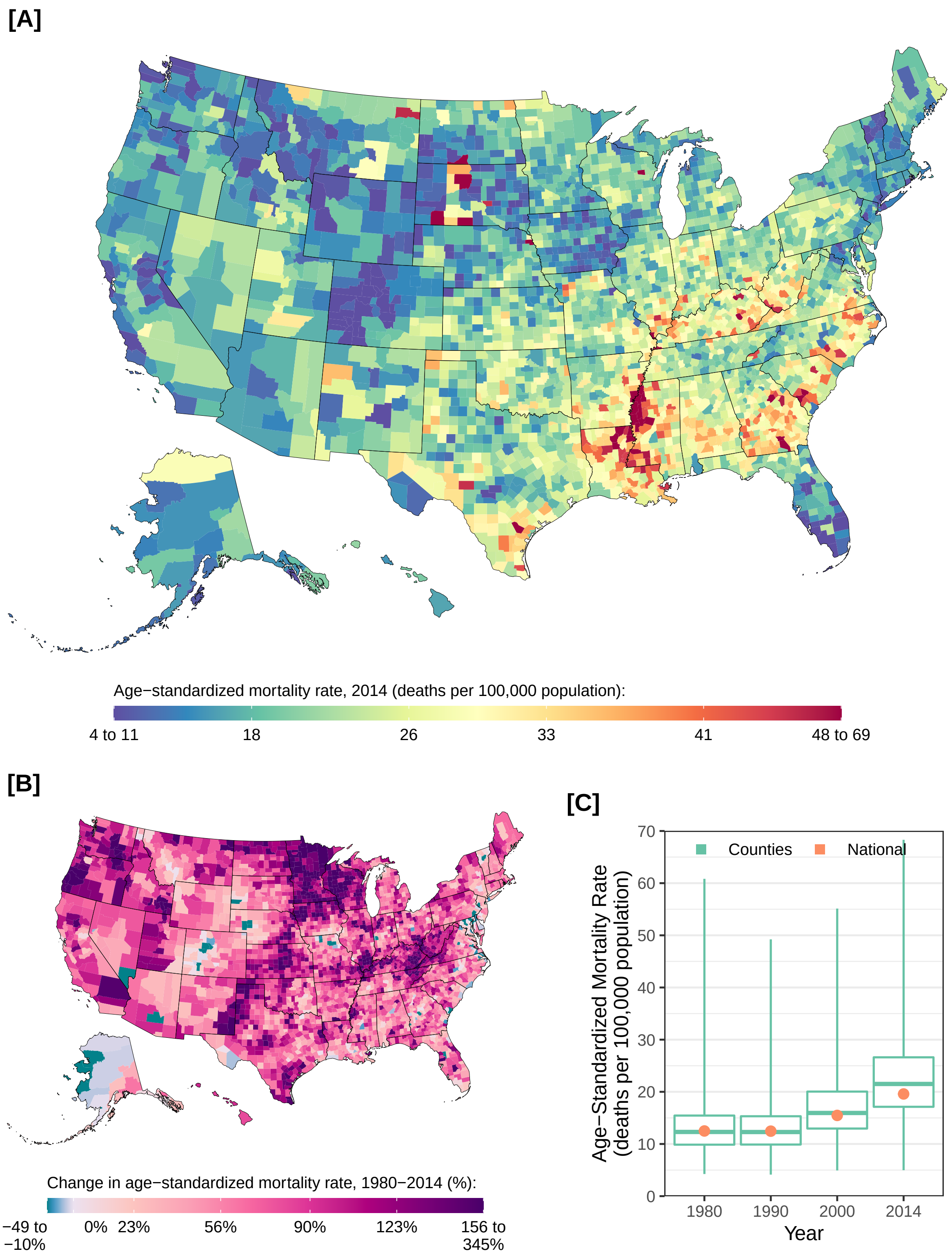

eFigure 4: County-level mortality among females from chronic kidney disease. [A] Age-standardized mortality rate in 2014; [B] Relative change in the age-standardized mortality rate between 1980 and 2014; [C] Age-standardized mortality rate in 1980, 1990, 2000, and 2014. In panel [C], the boxes indicate the 25th, 50th, and 75th percentile across all counties while the lines indicate the full range across counties and the dots indicate the national-level rate.

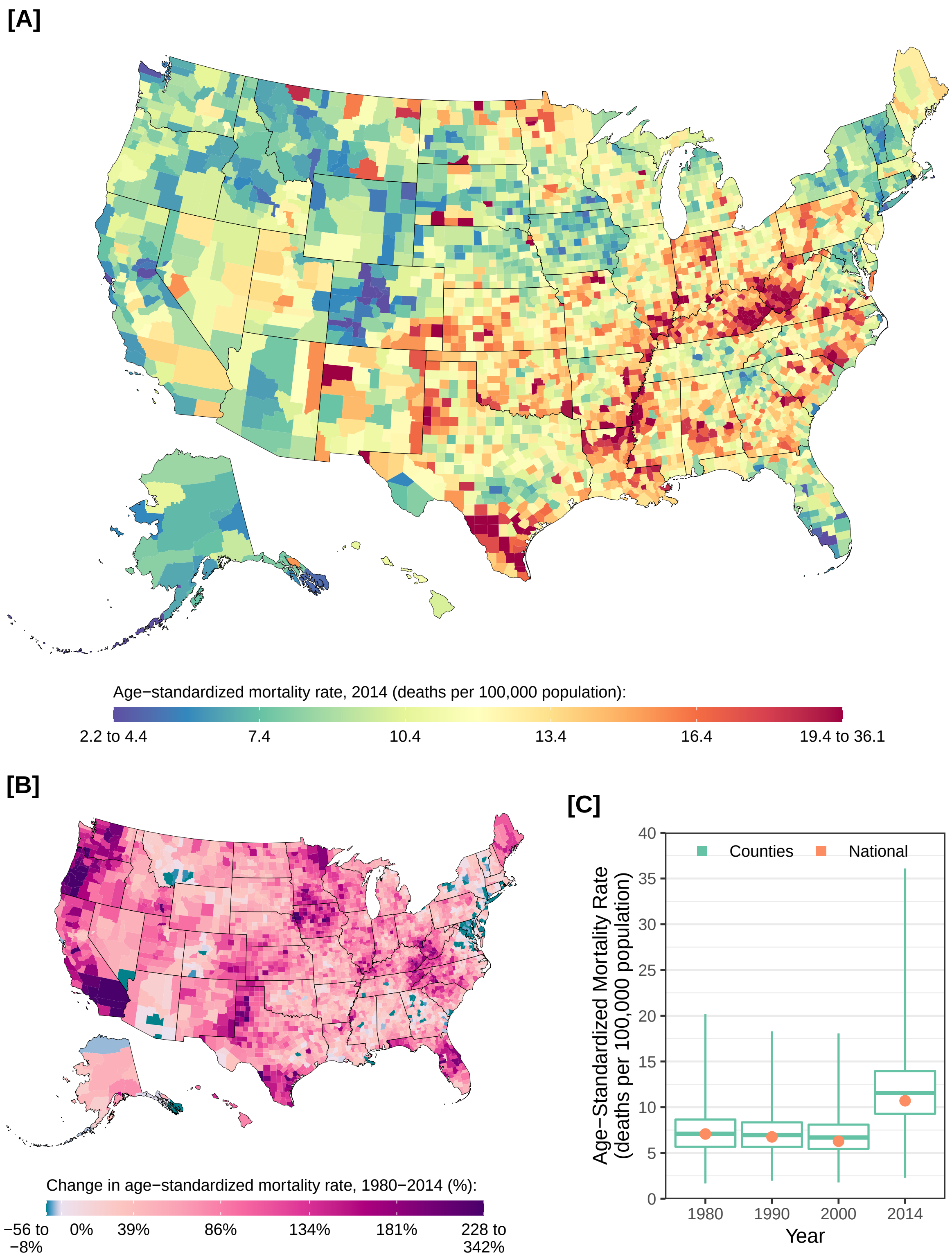

eFigure 5: County-level mortality among males from chronic kidney disease due to diabetes mellitus. [A] Age-standardized mortality rate in 2014; [B] Relative change in the age-standardized mortality rate between 1980 and 2014; [C] Age-standardized mortality rate in 1980, 1990, 2000, and 2014. In panel [C], the boxes indicate the 25th, 50th, and 75th percentile across all counties while the lines indicate the full range across counties and the dots indicate the national-level rate.

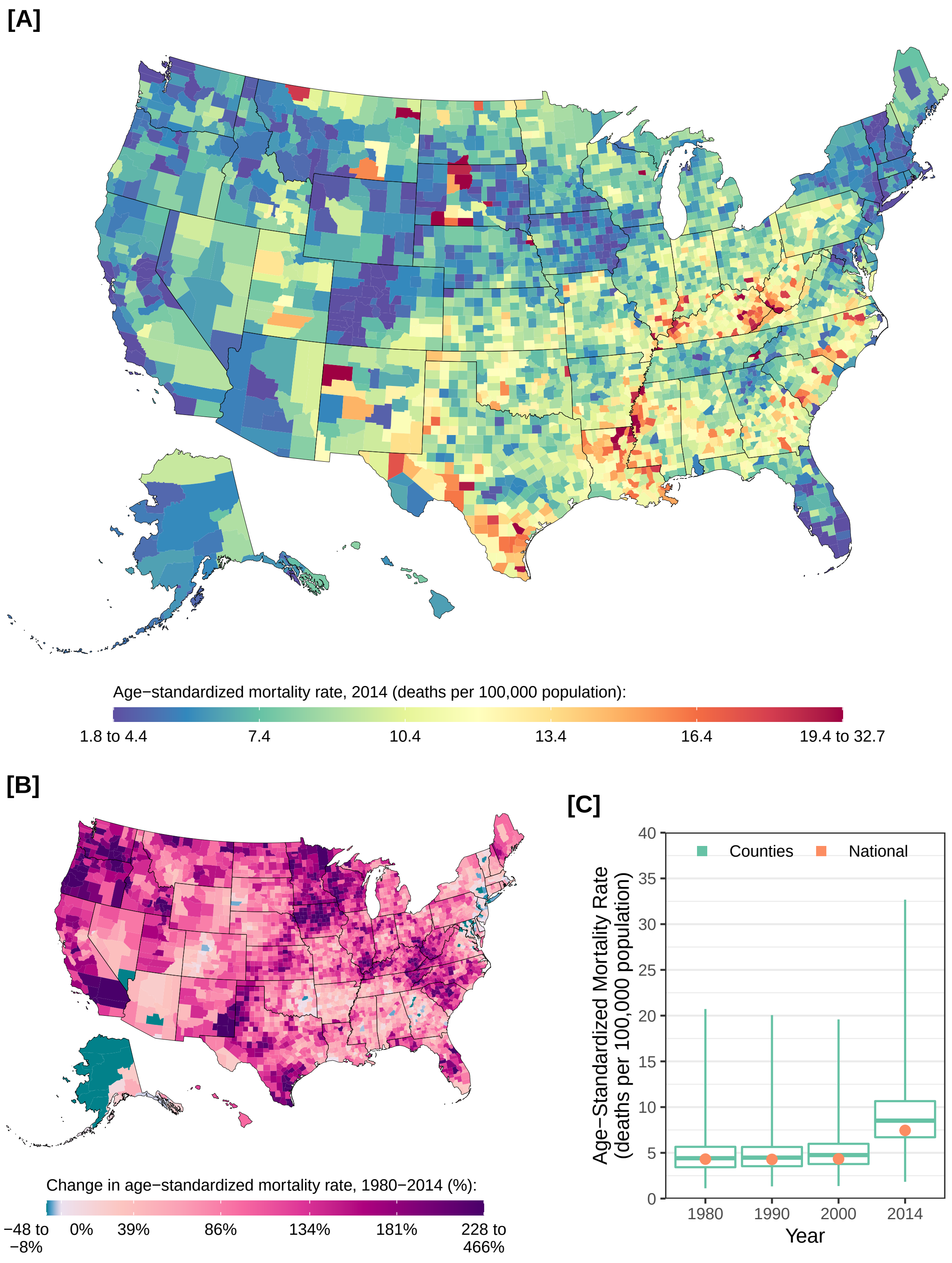

eFigure 6: County-level mortality among females from chronic kidney disease due to diabetes mellitus. [A] Age-standardized mortality rate in 2014; [B] Relative change in the age-standardized mortality rate between 1980 and 2014; [C] Age-standardized mortality rate in 1980, 1990, 2000, and 2014. In panel [C], the boxes indicate the 25th, 50th, and 75th percentile across all counties while the lines indicate the full range across counties and the dots indicate the national-level rate.

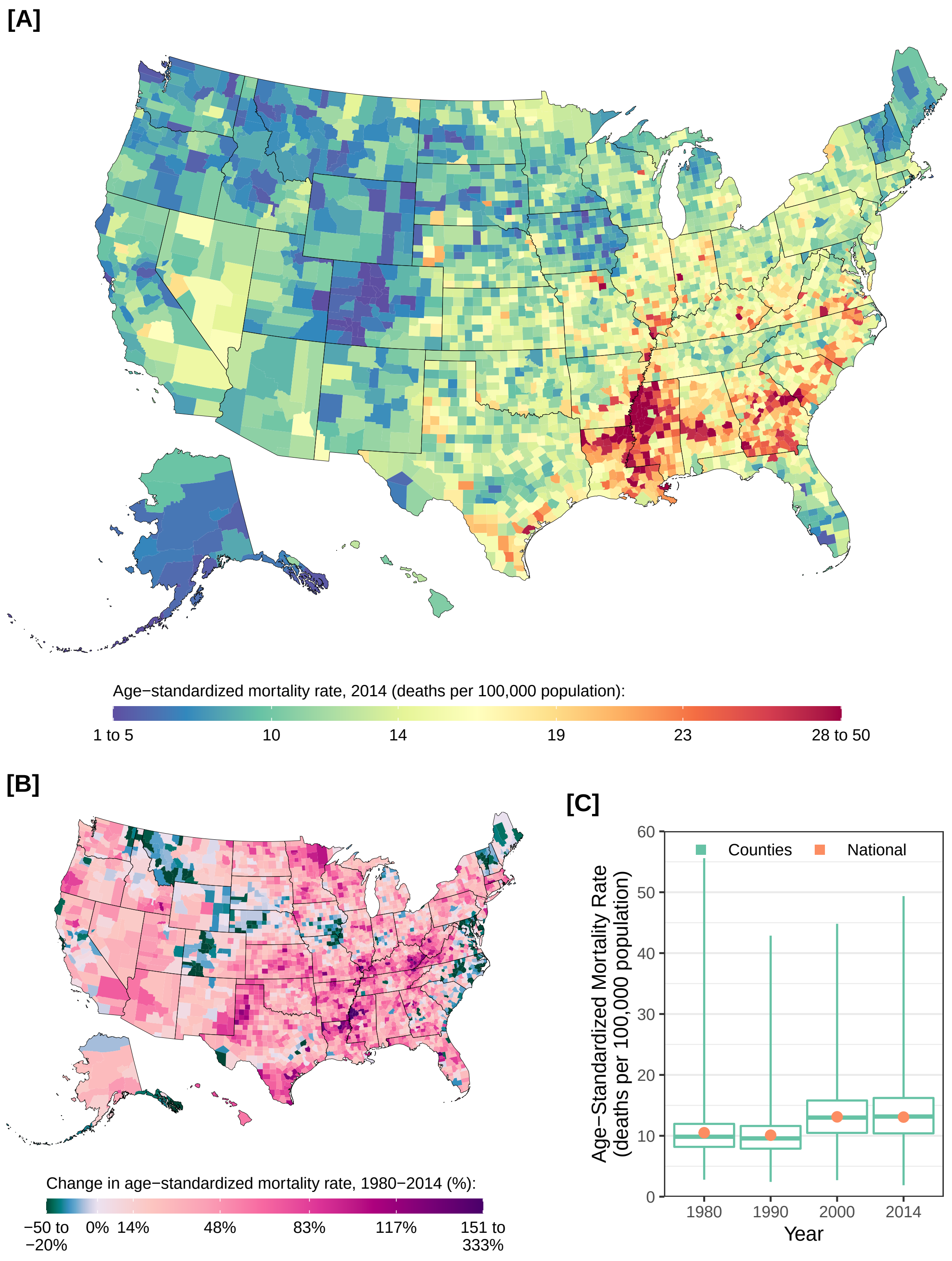

eFigure 7: County-level mortality among males from chronic kidney disease due to hypertension. [A] Age-standardized mortality rate in 2014; [B] Relative change in the age-standardized mortality rate between 1980 and 2014; [C] Age-standardized mortality rate in 1980, 1990, 2000, and 2014. In panel [C], the boxes indicate the 25th, 50th, and 75th percentile across all counties while the lines indicate the full range across counties and the dots indicate the national-level rate.

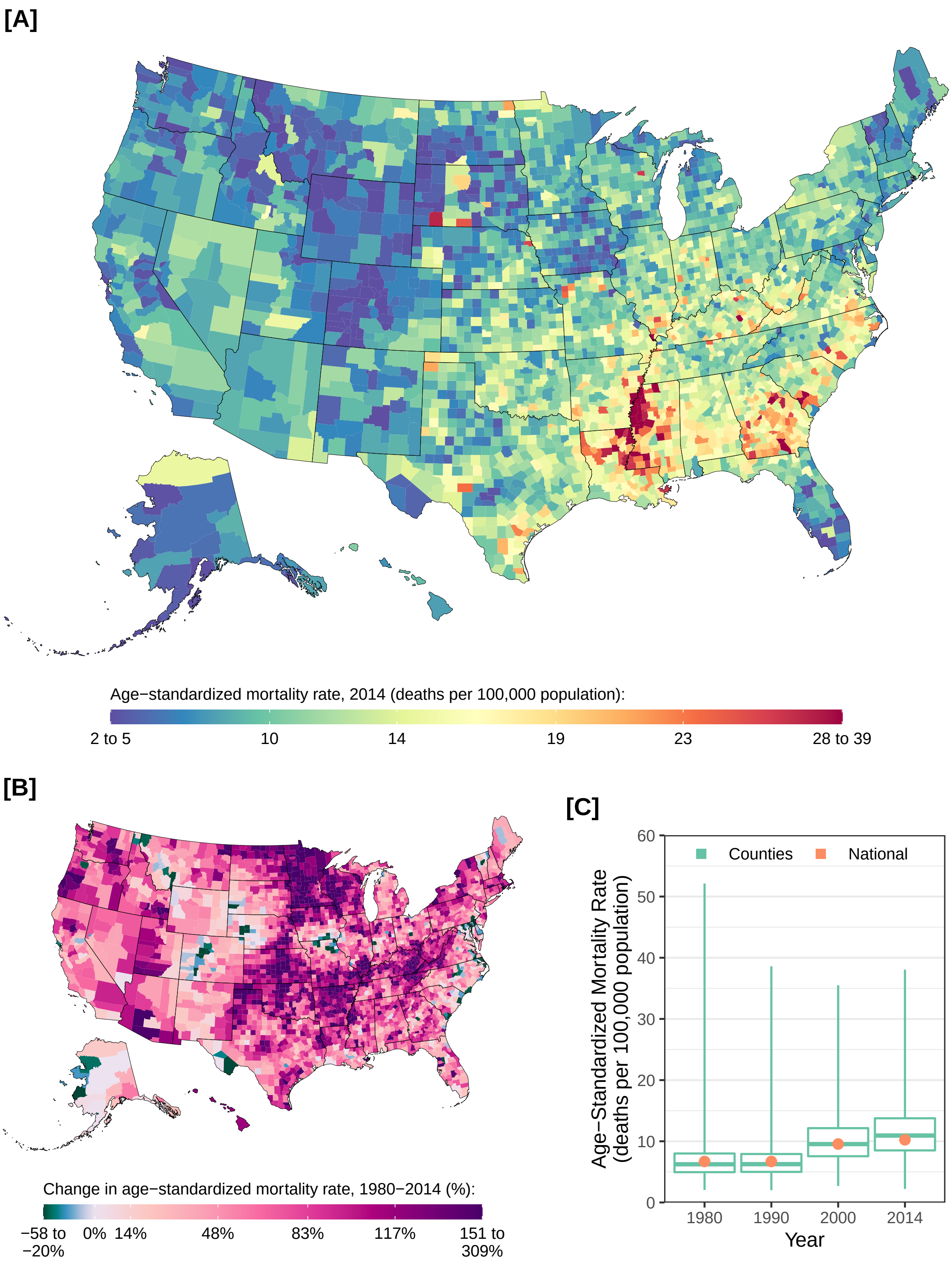

eFigure 8: County-level mortality among females from chronic kidney disease due to hypertension. [A] Age-standardized mortality rate in 2014; [B] Relative change in the age-standardized mortality rate between 1980 and 2014; [C] Age-standardized mortality rate in 1980, 1990, 2000, and 2014. In panel [C], the boxes indicate the 25th, 50th, and 75th percentile across all counties while the lines indicate the full range across counties and the dots indicate the national-level rate.

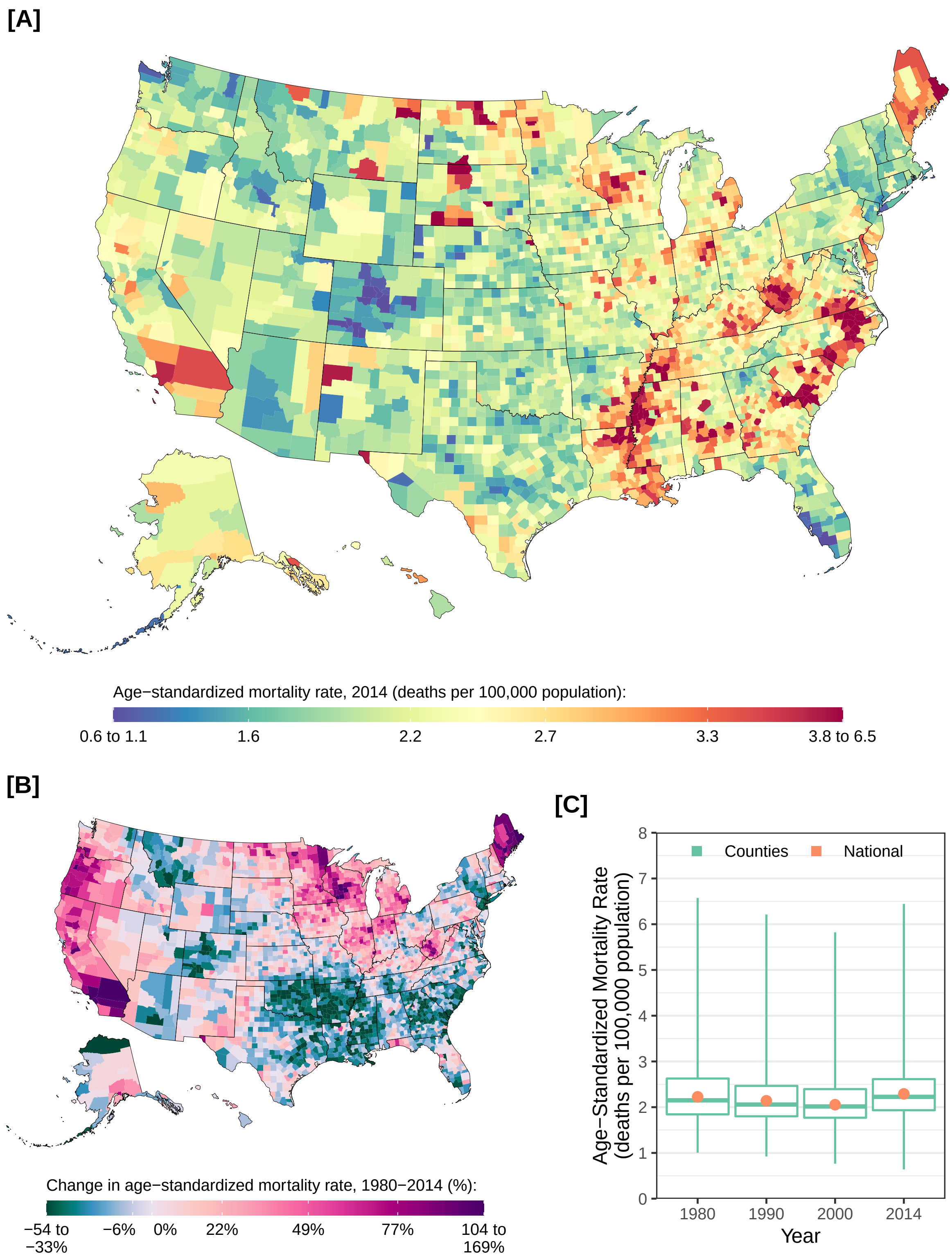

eFigure 9: County-level mortality among males from chronic kidney disease due to glomerulonephritis. [A] Age-standardized mortality rate in 2014; [B] Relative change in the age-standardized mortality rate between 1980 and 2014; [C] Age-standardized mortality rate in 1980, 1990, 2000, and 2014. In panel [C], the boxes indicate the 25th, 50th, and 75th percentile across all counties while the lines indicate the full range across counties and the dots indicate the national-level rate.

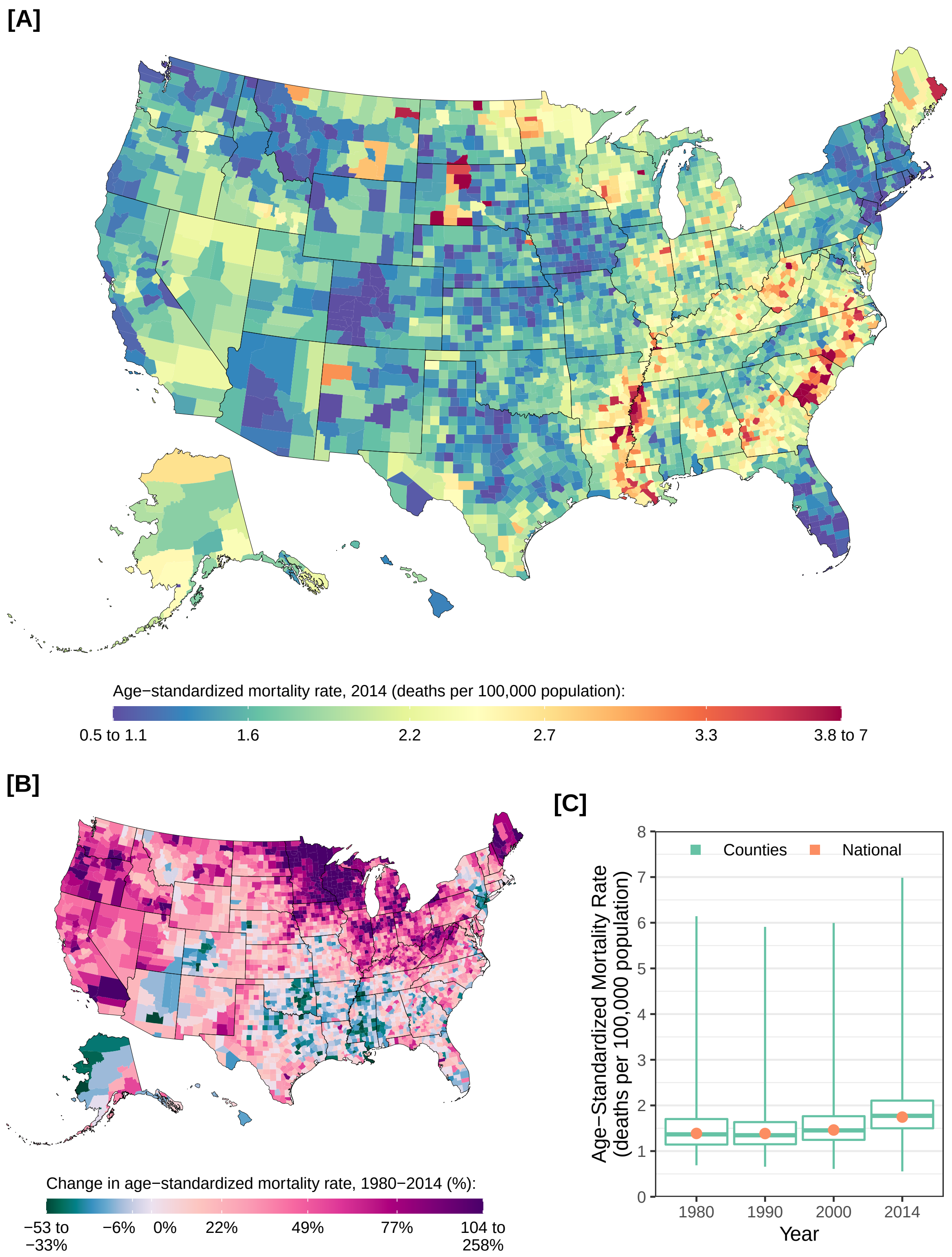

eFigure 10: County-level mortality among females from chronic kidney disease due to glomerulonephritis. [A] Age-standardized mortality rate in 2014; [B] Relative change in the age-standardized mortality rate between 1980 and 2014; [C] Age-standardized mortality rate in 1980, 1990, 2000, and 2014. In panel [C], the boxes indicate the 25th, 50th, and 75th percentile across all counties while the lines indicate the full range across counties and the dots indicate the national-level rate.

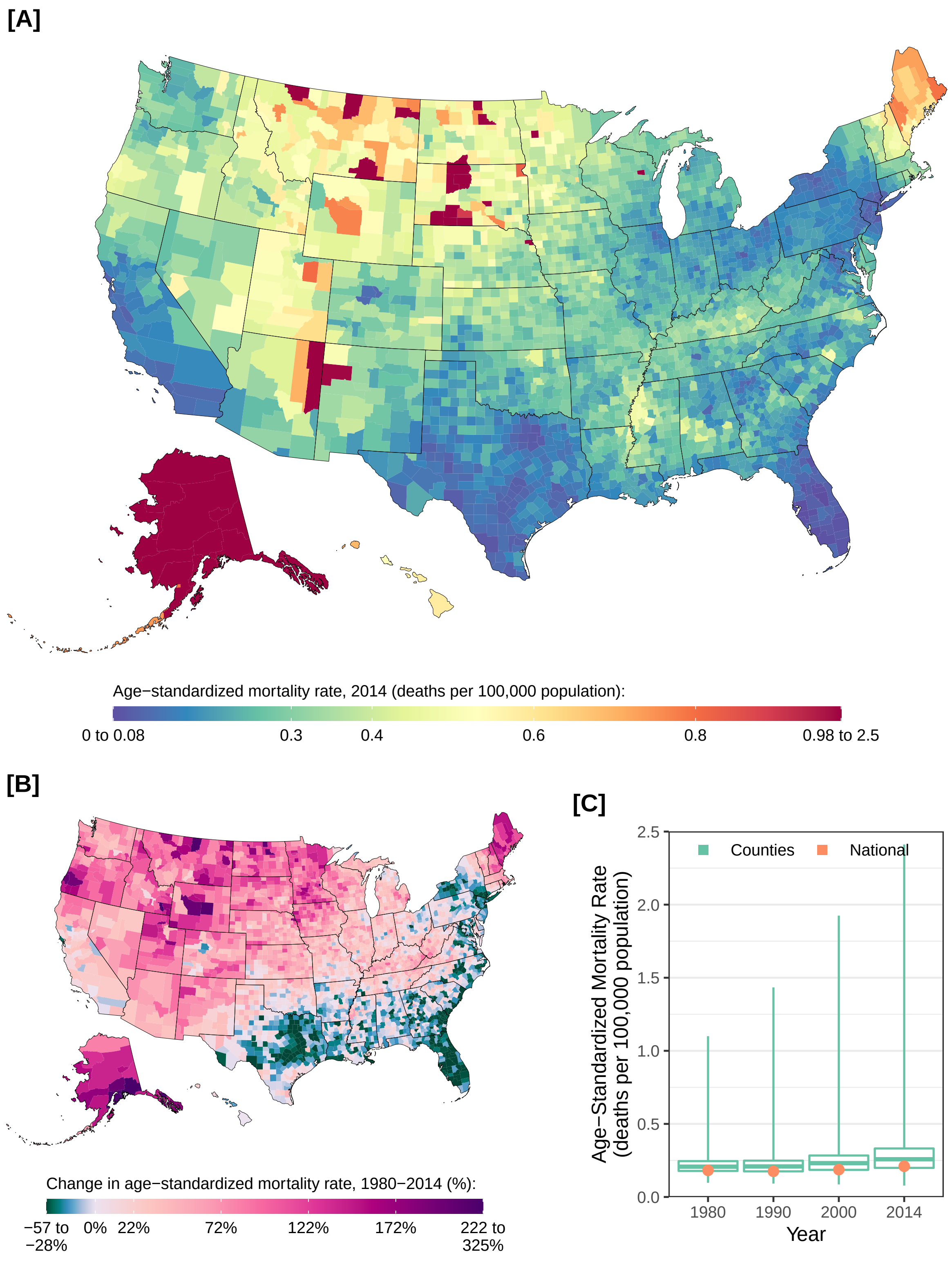

eFigure 11: County-level mortality among males from chronic kidney disease due to other causes. [A] Age-standardized mortality rate in 2014; [B] Relative change in the age-standardized mortality rate between 1980 and 2014; [C] Age-standardized mortality rate in 1980, 1990, 2000, and 2014. In panel [C], the boxes indicate the 25th, 50th, and 75th percentile across all counties while the lines indicate the full range across counties and the dots indicate the national-level rate.

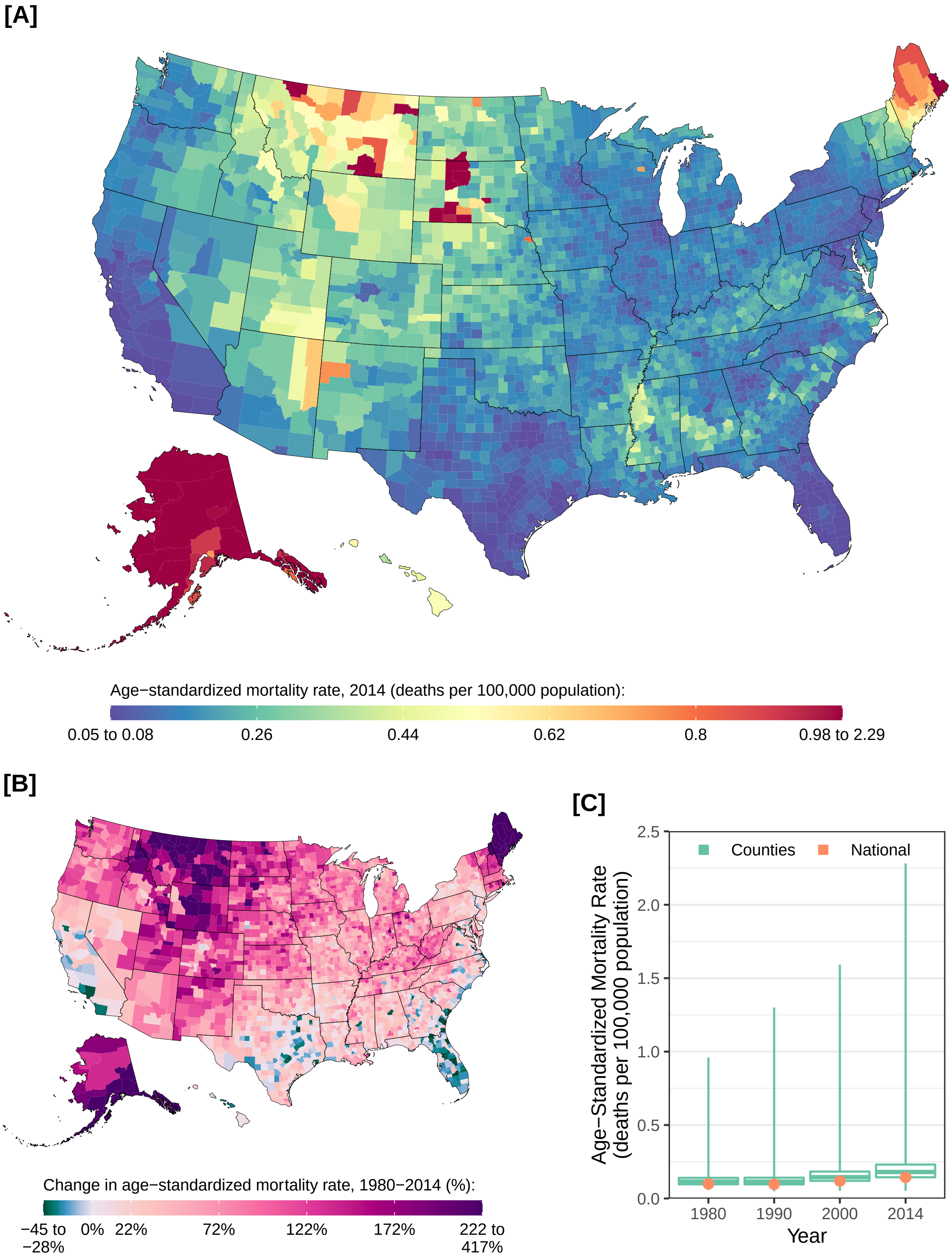

eFigure 12: County-level mortality among females from chronic kidney disease due to other causes. [A] Age-standardized mortality rate in 2014; [B] Relative change in the age-standardized mortality rate between 1980 and 2014; [C] Age-standardized mortality rate in 1980, 1990, 2000, and 2014. In panel [C], the boxes indicate the 25th, 50th, and 75th percentile across all counties while the lines indicate the full range across counties and the dots indicate the national-level rate.
